# The Formation of Hollow Trait in Cucumber (*Cucumis sativus* L.) Fruit Is Controlled by *CsALMT2*

**DOI:** 10.3390/ijms23116173

**Published:** 2022-05-31

**Authors:** Geng Zhou, Chen Chen, Xiaohong Liu, Kankan Yang, Chong Wang, Xiangyang Lu, Yun Tian, Huiming Chen

**Affiliations:** 1 Long Ping Branch, Graduate School of Hunan University, Changsha 410125, China; zhougeng@hnu.edu.cn (G.Z.); kankankkyang@163.com (K.Y.); 2 Hunan Vegetable Research Institute, Hunan Academy of Agricultural Sciences, Changsha 410125, China; ccad111188@163.com (C.C.); vseed@163.com (X.L.); 3 College of Bioscience and Biotechnology, Hunan Agricultural University, Changsha 410128, China; chongwang06@126.com (C.W.); xiangyangcn@163.com (X.L.)

**Keywords:** cucumber, fruit hollow trait, ventricular development, hollow gene

## Abstract

The hollow trait is crucial for commercial quality of cucumber (*Cucumis sativus* L.) fruit, and its molecular regulatory mechanism is poorly understood due to its environmental sensitivity. In the previous research, we obtained the hollow and the non-hollow materials of ecotype cucumbers of South China, which were not easily affected by the external environment through a systematic breeding method. In this study, first, we proposed to use the percentage of the hollow area as the criterion to compare the hollow characteristics between two materials, and to analyze the formation mechanism of early hollow trait from the perspective of cytology. The results showed that the hollow trait occurred in the early stage of fruit development, and formed with the opening of carpel ventral zipped bi-cell layer, which formed rapidly from 2 to 4 days, and then slowed to a constant rate from 14 to 16 days. Meanwhile, the different genetic populations were constructed using these materials, and fine mapping was performed by bulked segregant analysis (BSA) and kompetitive allele specific PCR (KASP) method. The *Csa1G630860* (*CsALMT2*), encoding protein ALMT2, was determined as a candidate gene for regulating the hollow trait in fruit. Furthermore, the expression profile of *CsALMT2* was analyzed by qRT-PCR and fluorescence in situ hybridization. The expression of *CsALMT2* had obvious tissue specificity, and it was abundantly expressed in the ovule development zone inside the fruit. In the hollow material of cucumber fruit, the expression of *CsALMT2* was significantly downregulated. The subcellular localization in tobacco leaves indicated that CsALMT2 was distributed on the plasma membrane. In conclusion, in this study, for the first time, we found the regulatory gene of hollow trait in cucumber fruit, which laid the foundation for subsequent research on the molecular mechanism of hollow trait formation in cucumber fruit, and made it possible to apply this gene in cucumber breeding.

## 1. Introduction

Cucumber (*Cucumis sativus* L., 2n = 2x = 14) is one of the vegetable crops with important agricultural, economic, and nutritional value in the world [1]. China is the largest cucumber producer and consumer in the world [2]. According to the Food and Agriculture Organization of the United Nations, the total production area of cucumber in the world in 2020 was 2.26 million hectares, and in China, it was 1.28 million hectares, accounting for about 56.6% of the world production. The total output of cucumber in the world was 91.25 million tons, while China was 72.83 million tons, accounting for 79.8% of the world production (http://www.fao.org/faostat/zh/#data/QC; accessed on 8 March 2022). The biological basis of cucumber fruit development and morphogenesis has always been a hot spot for research. Recent researches have mainly focused on fruit shape, fruit size, fruit quality, pulp and ventricular development, and peel and spine development [3,4]. However, pulp and ventricles are the major components of fruit, and there are more mature studies on ventricular development and genetic mechanism in tomato (*Solanum lycopersicum*) than in cucumber [5,6]. Due to defects during ventricular development, the hollow trait in fruit affects the commercial value and reduces the acquisition rate, and the larger hollowness reduces cucumber yield [7]. Therefore, there is an urgent need to solve the problem of fruit hollowness in the production and marketing of cucumber.

The hollow trait exists in many plant fruits, such as tomato, *Arabidopsis thaliana*, melon (*C. melo* L.), and cucumber, and is closely related to abnormal ventricular development during the formation of ovary tissue [6,8,9]. The ventricular tissue of tomato usually derives from the placenta, grows around the ovule, then the tissue expands and liquefies, and finally fills with a gelatinous substance to form a whole fruit [10,11]. The QTLs *fw2.2* and *fw3.2* that control cell division in carpels and peels [12,13] and the *fas* that encodes a transcription factor YABBY-like, which controls the size of tomato fruit by regulating the number of fruit ventricles [14], have been successfully obtained so far. Recent research has shown that the phenotype of all-flesh tomato was caused by structural variation composed of a 416-bp deletion that occurred in the cis-regulatory region of the *AFF* gene. In this phenotype, after the ovary tissue changed from gel-like material to solid tissue, the hollow trait of tomato fruit was formed because the ventricle could not be filled [6]. Cucumber fruit is a pepo that develops from the lower three carpels and receptacles of the ovary. On the basis of cross-sectional observations, the pulp of the cucumber fruit could be divided into three parts: exocarp, mesocarp and endocarp; it has also been observed that fruit hollowness mainly occurred in the endocarp [7]. Each ventricle in the endocarp is composed of a placenta, ventral carpel suture, ovule, and locule gel tissue [15]. Previous studies have mainly focused on the multi-ventricular trait. *CsCLV3* acts as a repressor, while *CsWUS* functions as an activator for carpel number variation in cucumber. *CsWUS* can directly bind to the promoter of *CsCLV3* and activate *CsCLV3* expression, while *CsCLV3* indirectly suppresses the activity of *CsWUS*. *CsFUL1^A^* has been shown to positively contribute to an increase in carpel number by directly binding to the promoter of *CsWUS* and promoting its expression. Auxin participates in carpel number regulation through interaction of *CsARF14* and *CsWUS* [16,17].

The hollow trait of cucumber fruit was first discovered by Hook (1876) in the mature fruit of Sikkim cucumber (*C. sativus* var. *sikkimensis*) [18]. The early field observations found that the formation of hollowness in cucumber fruit was related to factors such as variety, climate, and growth conditions [19]. Long-term water shortage in the soil, high soil nitrogen content, and abrupt changes in temperature during the fructification period were the major causes of hollow formation in cucumber fruit [20]. Recent research on hollow trait of Sikkim cucumber mature fruit has shown that the allele of *mfh3.1* in material ”WI7088D” could reduce the hollow size of fruit, while the allele of *mfh1.1* and *mfh2.1* could increase the hollow size of fruit [9]. The expression profile of *CsGID1a* has been reported to be closely related to locule formation in cucumber fruit. Silencing of cucumber *CsGID1a* might lead to abnormal development of carpels and ventricles and hollow trait in cucumber fruit, which would seriously affect the formation and development of seeds; however, the phenotype of hollow trait was significantly different from that of Sikkim cucumbers, which did not affect seed development [21]. Therefore, the mechanism of hollow formation in cucumber fruit deserves further exploration.

Due to the lack of experimental materials with low environmental impact and the stable hollow trait of cucumber varieties, it is difficult to develop insight into the genetic and regulatory mechanisms of the hollow trait in cucumber fruit. In this study, the cucumber variety H7 with non-hollow fruit and cucumber variety H6 with hollow fruit were selected as experimental materials, and fine mapping was carried out combining phenotypic analysis, cytological observation, genetic analysis, MutMap, and kompetitive allele specific PCR (KASP); the expression characteristics and location of the gene controlling hollow trait in cucumber fruit were also analyzed. The results might be helpful to understand the molecular mechanism of hollow formation in cucumber fruit, and provide useful information for cucumber breeding.

## 2. Results

### 2.1. Phenotypic Characterization of H6 and H7

The hollow traits of 29 new varieties of open field cucumbers (12 North China ecotype cucumber varieties and 17 South China ecotype cucumber varieties) were observed during the implementation of the key R&D project of the 13th five-year plan. The results showed that none of the North China ecotype cucumber varieties had hollow fruit, while 15 of the 17 South China ecotype cucumber varieties had varying degrees of fruit hollowness. The proportion of varieties with hollow fruit accounted for 88.24% (Table S1). 

**Figure 1:**
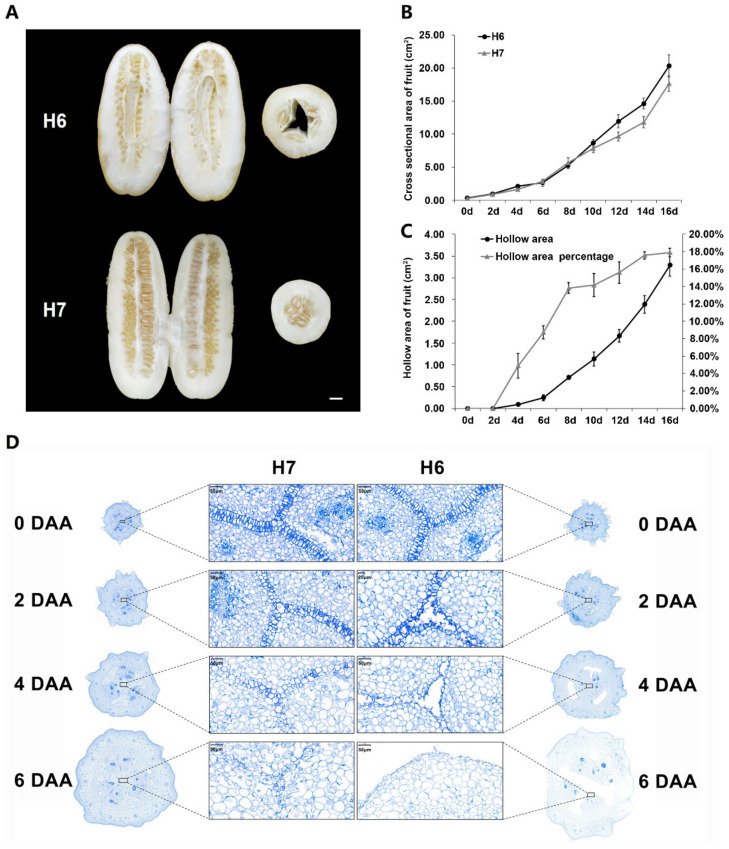
Identification of cucumber H6 and H7 hollow traits: (**A**) Internal phenotypic characteristics and hollow traits of H6 and H7 cucumber fruits, the fruits are at the maturity stage (Bars, 1 cm); (**B**) changes in the cross-sectional area between H6 and H7 from 0 to 16 days of fruit development; (**C**) changes of hollow area and percentage of hollow area in H6 from 0 to 16 days of fruit development; (**D**) cell structure of cross sections of H6 and H7 fruits between 0 and 6 days. DAA, day after anthesis.

Multigenerational breeding of ecotype cucumbers of South China was performed, and the sister lines H6 (hollow fruit) and H7 (non-hollow fruit) with stable hollow/non-hollow traits (Figure 1A) were obtained. In the commercial period, the fruit appearance of the two lines was basically the same, but the internal fruit structure was obviously different. The endocarp region of H6 had a large hollow, which did not affect seed development, while the endocarp area of H7 had full structure. There were hollow cucumber fruits with from three to five ventricles in the F_2_ generation, indicating that there was no inevitable relationship between the hollow trait and the number of ventricles. From the autumn of 2018 to the spring of 2020 in Hunan, the hollow trait in H6 and H7 fruit was observed for four times in the greenhouse. The ratio of hollowness in cucumber fruit was 100% in H6, while it was 0% in H7. During fruit development, the change trends were the same in fruit cross-sectional areas, but the cross-sectional area of H6 was slightly larger (Figure 1B). The hollow area of the fruit cross section in H6 increased exponentially over time, indicating that the hollow area size was related to fruit development, and the percentage of hollow area increased rapidly in the first eight days, then leveled off, and remained constant from 14 to 16 days of development. The change trend may be related to fruit development from the expansion stage to the mature stage (Figure 1C). In order to understand the changes of hollow trait in the early developmental stages of H6 and H7, the morphological changes from three days before flowering to six days after pollination were observed. There was no hollow phenomenon at the flowering and preflowering stages, and a hollow-like vascular bundle appeared at the end of ventral carpel suture after two days of pollination, and with the development of fruit, the hollow gradually expanded (Figure S1).

### 2.2. Cytological Observations of H6 and H7

The carpels of H6 and H7 fruits at different stages of early development were observed (Figure 1D). The ventral carpel sutures of H6 and H7 cucumbers on the day of flowering, i.e., 0 DAA (day after anthesis), were a tightly zipped bi-cell layer. The carpel ventral zipped bi-cell layer of H6 hollow material separated to form an early hollow phenomenon, which could not be distinguished by the naked eye before 2 DAA. From 2 DAA, the cells of H6 carpel expanded faster than those of H7, while the cell size of the carpel ventral zipped bi-cell layer did not change significantly (Figure S2). As time increased, the carpel ventral zipped bi-cell layer of H7 compressed, and became fuzzy and irregular, but did not divide to produce hollowness. The carpel ventral zipped bi-cell layer in H6 gradually separated into an irregular and smaller mono-cell layer. The hollow phenomenon was observed with the naked eye at the beginning of 4 DAA. It was speculated that the hollow phenomenon occurred at about 2 DAA of fruit development, and gradually formed with ventricle development, which pulled apart the carpel ventral zipped bi-cell layer.

### 2.3. Genetic Analysis of Hollow Traits in Cucumber Fruit

**Figure 2:**
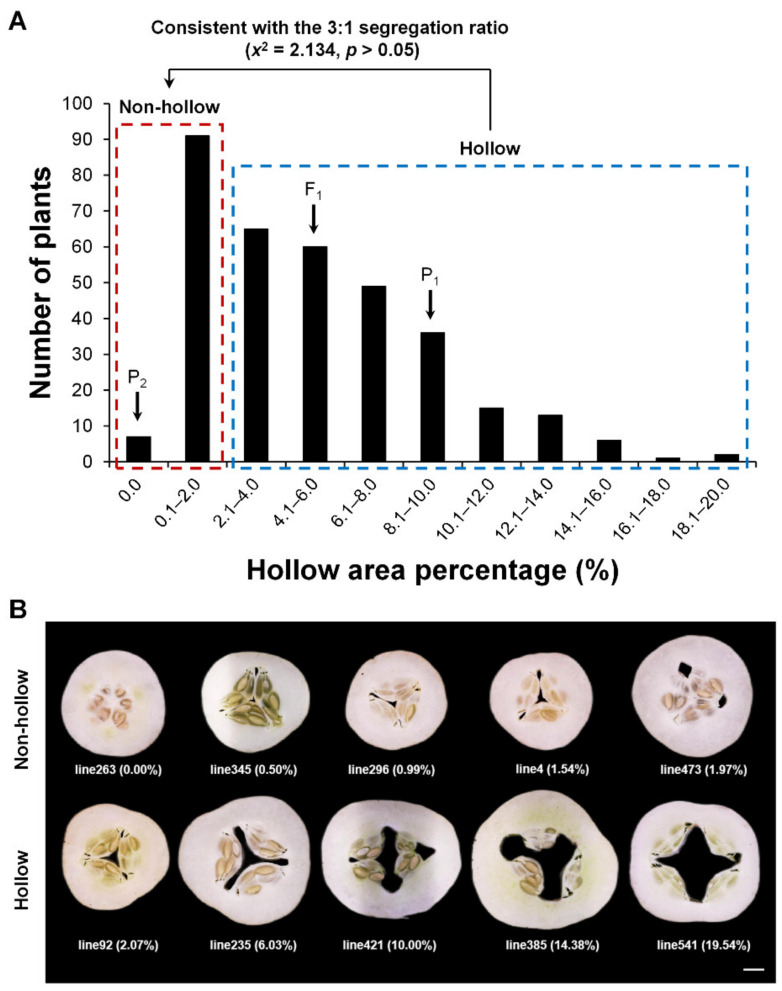
Frequency distribution of the hollow area percentage of cucumber fruit (**A**) and phenotypic identification (**B**) in F_2_. The numbers in brackets of the phenotypic identification picture represent the hollow area percentage.

In order to determine whether the hollow trait of cucumber fruit was controlled by a single gene or multiple genes, we analyzed the frequency distribution of hollow area percentage in the P_1_ (H6), P_2_ (H7), F_1_ (H6 × H7), and F_2_ (Figure 2A). The results showed that the average hollow area percentage of parent P_1_ (H6) was mainly between 8.1% and 10.0%. The trait of non-hollow material parent P_2_ (H7) was stable, and the hollow area percentage was 0%. The average hollow area percentage of F_1_ was distributed between 4.1% and 6.0%. The separated population F_2_ had a single peak with a left bias normal distribution, and the single peak distribution ranged from 0.1% to 2.0%, suggesting that there might be a major gene which was affected by the environment (Tables S2 and S3).

In 1965, the quantitative geneticist Falconer formally defined the threshold. The definition of the threshold, as the point on the scale of liability above which all individuals are affected and below which no one is affected, provides a fixed point for comparing different incidences of different populations or groups [22]. Using this method combined with phenotypic analysis in F_2_ (Figure 2B), it was found that with 2.0% as the threshold of hollow area percentage, 247 plants in F_2_ were hollow and 98 plants did not display hollowness. These findings were consistent with the 3:1 segregation ratio (*χ*^2^ = 2.134, *p* > 0.05), suggesting that the hollow trait of cucumber fruit was regulated by a single dominant gene in this population. Therefore, two pools were established to identify the causal gene using bulked segregant analysis (BSA) [23].

### 2.4. Whole Genome Resequencing Based on BSA Analysis

**Figure 3:**
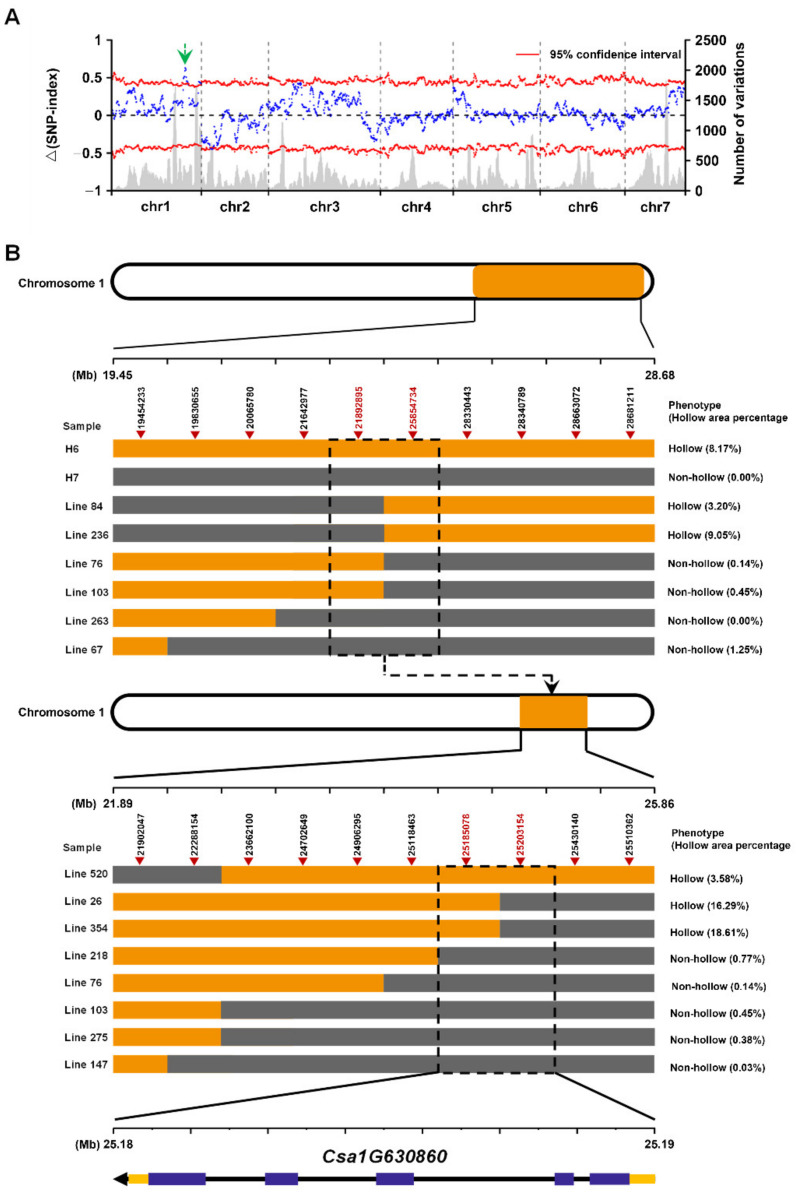
Fine mapping of the hollow gene in cucumber fruit based on genotypes and phenotypes of recombinant individuals: (**A**) Single nucleotide polymorphism (SNP)-index distribution. Blue lines indicate delta SNP-index, gray bars indicate the number of variations, the green arrow indicates the region on chromosome 1 with a SNP index >0.5; (**B**) genotyping analysis using the KASP technique in the F_2_ individuals. Gray bars are homozygous for the H7 allele and orange bars are homozygous for the H6 allele. Purple represents exons and yellow represents 5′-UTR and 3′-UTR.

Four cucumber DNA pools (H6, H7, F_2_ hollow, and F_2_ non hollow) were used for genome resequencing, and a total of 23.9 GB of sequence data were obtained. The GC content of samples ranged from 38.91% to 39.59%, and the Q30 value of all samples was greater than 90.29%, which indicated the output and quality of the sequencing data could fully meet the analysis requirements (Table S4). The reads were compared to the cucumber reference genome (ftp://www.icugi.org/pub/genome/cucumber/Chinese_long/v2/; accessed on 29 January 2021) using the BWA software (Table S5), the Samtools software was used to find the SNP sites in the whole genome, and 67,909 differential SNPs were finally obtained. We selected the different and homozygous SNP sites in the two parents, filtered out the SNP index deletion sites of the offspring, and found 3698 different SNPs, of which 2037 SNPs were identified on chromosome 1. There had been genetic linkage of the differential SNPs related to the target trait and its surrounding SNPs; we initially located the candidate region of the target gene between 24,747,234 and 28,340,789 bp on chromosome 1 (Figure 3A).

### 2.5. Fine Mapping and Identification of the Causal Gene

We constructed the F_2_ population with H6 (hollow fruit) and H7 (non-hollow fruit) as parents to verify whether the above regions were correct. Ten SNPs were mined based on the larger region containing the above region on chromosome 1. These SNPs were used to screen the recombinant individuals of the F_2_ generation population (345 recombinant individuals), and KASP was used for genotyping (Figure 3B). Firstly, the target gene was located between two SNPs (SNP21,892,895 and SNP25,854,734) on chromosome 1. Subsequently, in order to further narrow the candidate region of the target gene, we again designed 10 SNP markers in the 21,892,895–25,854,734 bp region of chromosome 1 for KASP genotyping validation. Finally, the target gene was located in the 25,185,078–25,203,154 bp region of chromosome 1. The above region was contained in the region Chr1: 24,747,234–28,340,789 bp, revealing the reliability of BSA analysis. The length of the region was 18,077 bp, and there was only one annotation gene *Csa1G630860* in the V2 version 9930 reference genome. The gene *Csa1G630860* encoded a type of widely studied transmembrane protein, aluminum-activated malate transporter (ALMT), and we named it *CsALMT2* for its homology to *AtALMT2* in *Arabidopsis*.

### 2.6. Sequence and Cis-Element Prediction of CsALMT2

**Figure 4:**
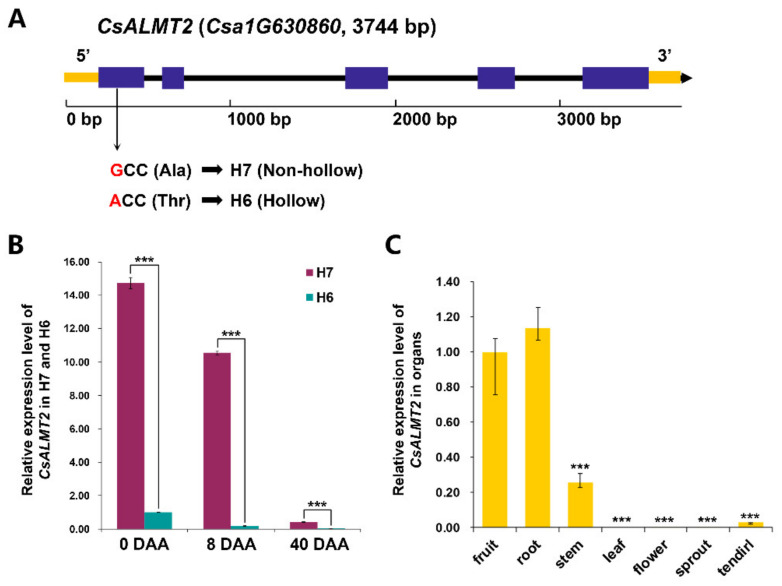
Gene structure and expression analysis of *CsALMT2*: (**A**) Gene structure of the predicted *CsALMT2*. Purple represents exons, solid lines represent introns, and yellow represents 5′-UTR and 3′-UTR; (**B**) relative expression levels of *CsALMT2* in H7 and H6 fruit at different growth stages. Bars are means of three replicates ± SEM. Triple asterisk, *p* < 0.001; (**C**) relative expression levels of *CsALMT2* in H7 different tissues. Bars are means of three replicates ± SEM. Triple asterisk, *p* < 0.05.

**Figure 5:**
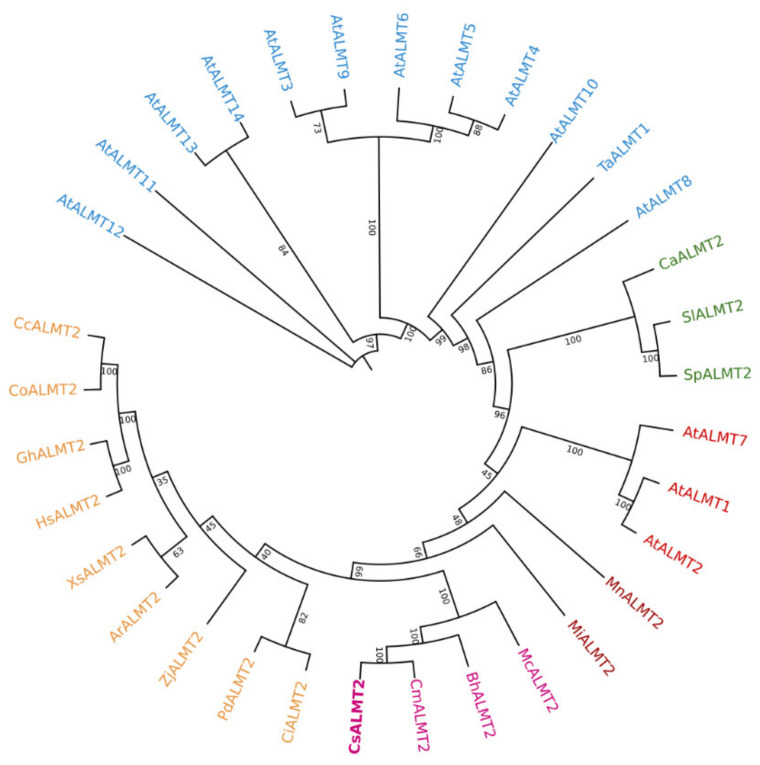
Phylogenetic analysis of CsALMT2. ALMT2 protein in different species and phylogenetic tree of other members of the ALMT family in *Arabidopsis*. The phylogenetic tree is generated using the maximum likelihood method in Omicshare Tools online software, and the inferred phylogeny is tested through the guided analysis of 1000 duplicate datasets. The number displayed at the tree fork represents the frequency of occurrence in all boot iterations performed.

The full-length of *CsALMT2* genes in H6 and H7 were amplified and sequenced, respectively. The sequence comparison showed that *CsALMT2* had four SNPs in the exon of H6 and H7, of which three were distributed on exon 1 and only one on exon 2. The sequences of the remaining three exons in H6 and H7 were completely identical. Further analysis revealed that only the transversion G82A (SNP28756924) on exon 1 of the four SNPs resulted in the amino acid changing from alanine to threonine (A28T) (Figure 4A). CsALMT2 consisted of 454 amino acids. The results of phylogenetic analysis reflected that CsALMT2 clustered together with ALMT2 in other species, but was distant from other members of the ALMT family. It was closest to CmALMT2 and BHALMT2 in the evolutionary tree (Figure 5). In the local multiple sequence alignment, there were no amino acid changes from A to T in the same position of melon (*C. melo*), wax gourd (*Benincasa hispida*), and the sequenced reference genome varieties (Gy14, PI183967, and 9930), suggesting that the amino acid changes at this position affected the protein function, and led to the hollow phenomenon of cucumber (Figure S3). The sequence domain analysis of CsALMT2 protein found that the predicted protein domains consisted of four categories, including four cytoplasmic domains, three non-cytoplasmic domains, six transmembrane (TM), and six TM helix. The change of A28T occurred in the cytoplasmic domain (Figure S4). In addition, we also analyzed the promoter region of *CsALMT2* and detected the light response elements, MYB binding sites involved in drought induction, defense and stress response elements, and a large number of hormone response elements (abscisic acid response element, gibberellin response element, auxin response element, salicylic acid response element, etc.) (Table S6). These results reflected that the expression of *CsALMT2* was vulnerable to environmental changes.

### 2.7. Expression Characteristic Analysis of CsALMT2

We also investigated the expression profiles of *CsALMT2* in H6 and H7 at different stages (Figure 4B). The results showed that the expression level of *CsALMT2* in H7 was significantly higher than that in H6 at each stage. The expression level of *CsALMT2* in non-hollow material H7 was 14.7 times that of hollow material at 0 DAA, while the expression level of *CsALMT2* in non-hollow material H7 was 59.3 times that of hollow material at 8 DAA. Although the expression levels of *CsALMT2* were very low in both materials at 40 DAA, the expression level of *CsALMT2* in non-hollow material H7 was 69.7 times higher than that of hollow material. In both H6 and H7, the expression levels of *CsALMT2* were significantly decreased over time. The expression level of *CsALMT2* in non-hollow material H7 at 0 DAA was 1.4 times that of 8 DAA, while the expression level of *CsALMT2* in hollow material H6 at 0 DAA was 5.5 times that of 8 DAA. The expression level of *CsALMT2* in non-hollow material H7 at 8 DAA was 25.3 times that of 40 DAA, while the expression level of *CsALMT2* in hollow material H6 at 8 DAA was 29.7 times that of 40 DAA. As compared with H7, the expression level of *CsALMT2* in H6 decreased more rapidly with time. In order to understand the spatial expression characteristics of *CsALMT2*, we detected the expression levels of *CsALMT2* in fruits, roots, stems, leaves, flowers, sprouts, and tendrils of H7 (Figure 4C). The results suggested that *CsALMT2* exhibited a tissue specific expression, and the expression levels in roots and fruits were much higher than that in other tissues.

### 2.8. Fluorescence In Situ Hybridization

**Figure 6:**
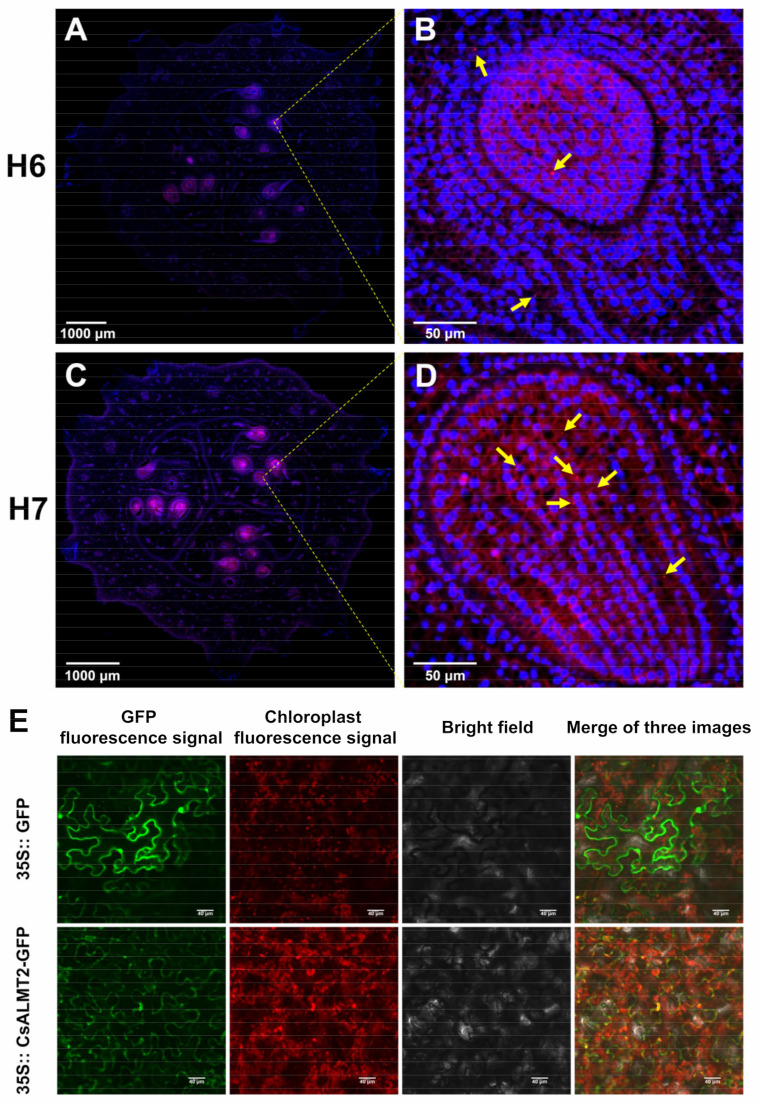
Fluorescence in situ hybridization analysis and subcellular localization in *CsALMT2*: (**A**,**B**) Images of H6 fruit at 0 d under a 1× and 30× fluorescence microscope, respectively; (**C**,**D**) images of H7 fruit at 0 d under a 1× and 30× fluorescence microscope, respectively. Blue light represents the nucleus, red light is the positive expression of fluorescein labeling. Yellow arrows represent positive expression of *CsALMT2*. (**E**) subcellular localization of CsALMT2 in tobacco cells.

Fluorescence in situ hybridization (FISH), an in situ hybridization technology using fluorescence signal detection probe, has been the most important technology in plant cytogenetics [24]. The FISH discovered that the number of red specific bright spots in early fruit of H7 was significantly more than that of H6, mainly concentrated in the ovule development area of the fruits (Figure 6A–D). The differences reflected the different hollow traits in H7 and H6 fruits, and were also consistent with the expression characteristics of *CsALMT2*. In addition, the majority of specific bright red spots were separated from the bright blue spots representing nuclei, suggesting that *CsALMT2* might function in the cell membrane or cytoplasm.

### 2.9. Subcellular Localization of CsALMT2-Encoding Protein

We used WoLF PSORT and CELLO online softwares to predict the subcellular localization of CsALMT2. The prediction results of WoLF PSORT showed that among the 14 nearest neighbors, seven were located in the plasma membrane, five were located in the vacuole, and one was located in the endoplasmic reticulum and one was located in the Golgi apparatus. The CELLO prediction results showed that CsALMT2 was located on the plasma membrane with the reliability value of 4.256, which was much higher than that located on other structures. In order to understand the specific localization of CsALMT2 protein in cells, first, we used pCambia1301-JC-GFP as the vector skeleton and inserted the gene *CsALMT2* into the vector. Subsequently, *Agrobacterium tumefaciens* carrying 35S: CsALMT2 GFP vector was transiently transferred to tobacco, cultured under weak light for two days, and then observed and photographed by a laser confocal microscope (Nikon C2-ER). The results showed that the expression intensity of ALMT2-GFP was weaker than that of the GFP control, but the GFP signals were distributed over the structures of cell membrane and plasma membrane, and possibly coincided with the chloroplast (Figure 6E).

## 3. Discussion

The shape and internal structure of cucumber fruit are the decisive characteristics for market orientation in commercial production, and from the perspective of crop breeding, these characteristics also represent fruit quality and yield traits in horticulture [4]. Research on the shape characteristics in cucumber fruit is relatively mature, but there has been less research on the fruit internal structure. The problem of hollow trait, which is a typical internal structure trait of cucumber fruit, originated from the production practice. In the early years, the hollow defect of cucumber fruit resulted in production processing losses of nearly 30% in Poland [20]. An investigation of 17 new South China ecotype cucumber varieties found that more than 88% of the varieties had hollow fruit, which suggested that the hollow trait of cucumber fruit was common in South China cucumbers (Table S1). Because the period of hollow formation in cucumber fruit matches the period of market fruit, the period for an investigation is relatively late, requires hard work, and is time-consuming; it is difficult to detect the trait at an early stage of cucumber growth. In addition, because it is difficult to distinguish the appearance of hollow fruits from non-hollow fruits, consumers who buy hollow fruits stop purchasing due to quality problems, which eventually leads to a more obvious drop in sales and prices. Although the hollow trait causes serious harm to cucumber production, there is still no further research on its genetic basis and molecular mechanism due to the lack of genetically stable cucumber materials.

Through systematic breeding and a multigenerational investigation of population hollowness, we have bred cucumber materials H6 (hollow fruit) and H7 (non-hollow fruit) with stable hollow traits. Based on this, in this study, we further improved the judgment standard for hollow trait. Wang et al., in a study on the hollow trait of Sikkim cucumber, introduced two standards, namely, immature fruit hollow size and mature fruit hollow size, to measure the degree of hollowness in fruit [9]. In this study, the percentage of hollow area was used as the measurement standard of hollow size, which reduced the error caused by the later expansion of cucumber fruit, and also improved the accuracy of judgment and advanced the hollow trait investigation time. From the results of the trait investigation, hollow trait slightly increased the cross-sectional area of fruit (Figure 1B). The formation of fruit hollowness occurred in the early stage of fruit development (Figure 1D and Figure S1), and the hollowness formed rapidly from two to four days, and then gradually slowed down to a constant rate by 14 to 16 days (Figure 1C). The result was consistent with a previous study, that is, hollowness appeared in young cucumber fruits with a fruit diameter of 1.5 cm, which increased with fruit development [20]. In addition, it had been observed that the ovary shape of cucurbit crops (cucumber, squash, zucchini, melon, etc.) was positively correlated with the final fruit shape, which was mainly determined before flowering [4,25]. During the development of melon fruit, with an increase in fruit diameter, the ovary fruit shape index was higher than that of mature fruit, and the final fruit shape reached a stable level about 15 days after flowering [26]. The growth cycle of cucumber fruit is as follows: the slow growth stage of fruit length and diameter growth within 0–4 days after flowering, the rapid growth stage after 4 days, the arrest of growing after 16 days, and the mature stage [27]. We speculate that the hollow trait, as an internal structural trait of fruit, may be maintained for a period similar to external development.

The development of cucumber fruit is mainly due to changes in cell number and volume [28,29,30]. Elkner (1982) previously proposed that the uneven enlargement of adjacent cells in the carpel suture of cucumber fruit generated strong mechanical tension between them, which could be responsible for the separation of carpel suture after cell rupture [20]. This view was consistent with our slice observation, in which the carpel cells of H6 expanded faster than those of H7 after two days of flowering, the separation of ventral carpel suture bi-cell layer was similar to zipper, and it was easier to separate for the ventral carpel suture bi-cell layer near the epicarp, though we tended to hypothesize that the separation of the ventral carpel suture bi-cell layer was caused by the tension caused by the development and atrophy of each locule.

Malic acid, as a central metabolite, has been shown to be associated with various metabolic pathways in plants, and to play crucial roles in various functions, such as participating in ATP production, fatty acid metabolism, acting as a temporary carbon pool, and pH regulation [31]. The genes encoding ALMT have been identified in many plant species, and the ALMT family has been proved to be central to physiological processes [32]. In this study, the South China ecotype cucumber fruit hollow material H6 and non-hollow material H7 were used for fine mapping through BSA-seq and KASP genotyping, and the *Arabidopsis ALMT2* homologous gene *CsALMT2* (*Csa1G630860*) was finally identified as a candidate gene to regulate the hollow trait in fruit (Figure 3). Sequencing analysis revealed that the transversion of G82A on exon 1 of the gene resulted in an amino acid change from alanine to threonine (A28T) (Figure 4A). The phylogenetic analysis indicated that CsALMT2 was most closely related to CmALMT2 and BhALMT2. The distance between CsALMT2 and AtALMT2 was closer than that of other members of the *Arabidopsis* ALMT family (Figure 5). It was predicted that CsALMT2 belongs to transmembrane protein, and contained six transmembrane domains (Figure S4). The *ALMT* gene family encodes anion transport proteins, and also regulates the transmembrane permeability of organic acids in plants [33]. ALMT has multiple potential roles in plant metabolism, including regulation of organic acids in fruits, movement of guard cells, and induction of plant tolerance to aluminum stress [34]. It was initially found that *TaALMT1* could confer resistance to aluminum toxicity in plants [35,36], and the extracellular hydrophilic carboxyl-terminal domain could regulate the activity of TaALMT1 [37]. Later, it was proven that *BnALMT1* and *BnALMT2* could also enhance the aluminum resistance in plant cells [38]. *AtALMT1* conferred aluminum tolerance in Arabidopsis [39,40]. AtALMT9 was a malate-activated vacuole chloride channel required for stomatal opening in *Arabidopsis* [41], meanwhile, AtALMT9 could also affect the content of malic acid in cells [31]. AtALMT12 has been reported to play an important regulatory role in controlling stomatal guard cells in *Arabidopsis* [42]. Seventeen *HbALMT* genes have been identified from the genome of the rubber tree, most of which responded to aluminum stress [43]. In maize, *ZmALMT1* has been shown to encode a plasma membrane transporter, which could mediate the outflow and inflow of selective anions [44], while *ZmALMT2* released malic acid in roots, and also played an important role in the transport of organic acids in xylem [45]. The barley gene *HvALMT1* has been shown to encode anion channels in guard cells and some root tissues, which promoted stomatal closure by affecting malic acid release in guard cells [46]. The rye gene *ScALMT1* has been shown to confer aluminum tolerance to plants [47]. *GmALMT1* in soybean was reported to be involved in regulating malic acid exudation, which improved the adaptability to acidic soil in plants [48]. Previous studies in tomato have found that *SlALMT3* participated in the crosstalk regulation mechanism between aluminum and jasmonic acid in the process of inhibiting root growth. *SlWRKY* may participate in this crosstalk reaction as an upstream regulator of *SlALMT3* [49]. *SlALMT4* and *SlALMT5* has been shown to be involved in malic acid transport. *SlALMT9* could regulate the secretion of malic acid in tomato and promoted the accumulation of malic acid in fruit [50,51]. Although the functional importance of the *ALMT* gene has been fully confirmed in many species, *ALMTs* have been reported to be involved in different biological processes of the plant life cycle, and to exert multiple functions in different species. *ALMT* has rarely been reported in Cucurbitaceae until now, the latest research on *WmALMT*-3 in watermelon pulp found that it may be involved in the regulation of malic acid and that it accumulates in vacuoles during watermelon fruit development [52]. According to the results of *ALMTs* in the reference, we predict that *CsALMT2* may affect the hollow trait by regulating the distribution of organic acids in cucumber; however, the relevant experimental data and evidence still need to be accumulated.

The expression level of *CsALMT2* in H7 was significantly higher than that in H6 at each stage, and the tissue expression levels varied in tissues (Figure 4). The results of the FISH showed that *CsALMT2* was widely distributed in the ovule development area (Figure 6A–D). The *HvALMT1* has been shown to play a variety of roles in seed development and grain germination [46], and overexpression of *SlALMT5* could increase the content of malic acid in mature tomato seeds [50]. We speculated that *CsALMT2* might affect the hollow trait in cucumber fruit, and also the content of malic acid in fruit and seeds. In addition, the CsALMT2 was predicted to localize to the plasma membrane by WoLF PSORT and CELLO. This study also proved that CsALMT2 was located in cell membrane and plasma membrane by subcellular localization of tobacco transient analysis, which was in accordance with previous predictions (Figure 6E). The ALMT proteins are mostly located in cell membrane, vacuolar membrane, and plasma membrane [32]. ALMT is located in plasma membrane in wheat, *Arabidopsis*, maize, and rape seed [35,39,44]. The latest study predicted that 24 EjALMT proteins in loquat were also located in the plasma membrane [34]. At present, the mechanism of how ALMT participates in the regulation of cucumber fruit development is still unclear and needs further research, which would help to analyze the mechanism of hollow trait formation.

## 4. Materials and Methods

### 4.1. Plant Materials and Crossing

#### 4.1.1. Plant Materials

All cucumber materials in this study were provided by the cucumber research group of the Hunan Vegetable Research Institute. Cucumber varieties ♀j and TS-1 were selected as parents for hybridization. The ♀j variety was the South China ecotype cucumber, with non-hollow fruit, gynoecious type, medium parthenocarpy ability, and the commercial fruit length is about 25 cm. The TS-1 variety was the South China ecotype cucumber, with non-hollow fruit, monoecious type, strong parthenocarpy ability, and the commercial fruit length is about 25 cm. The sister lines H6 (large hollow, gynoecious type, strong parthenocarpy ability, and the commercial fruit length is about 12 cm) and H7 (non-hollow, gynoecious type, weak parthenocarpy ability, and the commercial fruit length is about 12 cm) with stable hollow/non-hollow trait were obtained in the seventh generation by systematic breeding method. The experiments were carried out in the greenhouse of the Hunan Vegetable Research Institute from August 2016 to June 2019.

#### 4.1.2. Construction of Genetic Population

In the autumn of 2019, a hybrid combination H6 × H7 was created with cucumber H6 (hollow fruit) as the female parent and H7 (non-hollow fruit) as the male parent in the greenhouse of the Hunan Vegetable Research Institute. The F_1_ seeds were sown in the spring of 2020, the F_2_ was obtained by selfing at the flowering stage, then four genetic generations, i.e., P_1_, P_2_, F_1_, and F_2_, were constructed, the seeds of which were sown in the autumn of 2020. The sample sizes of lines in each population were 19 for P_1_, 20 for P_2_, 20 for F_1_, and 345 for F_2_. The seedlings were raised in seedling tray and transplanted in the greenhouse. Genetic analysis of each population was carried out in the greenhouse.

#### 4.1.3. Identification of Hollow Trait of New Open Field Cucumber Varieties in China

The hollow traits of 29 new varieties of open field cucumber (12 North China ecotype cucumber varieties and 17 South China ecotype cucumber varieties) were investigated in the spring of 2019. ZhongNong38, ZhongNong48, ZhongNong106, 18C54, and 18C56 were provided by the Institute of Vegetables and Flowers, Chinese Academy of Agricultural Sciences; YD109 and YD110 were provided by the Yangzhou University; HN1, HN2, HN3, and HN4 were provided by the Hunan Vegetable Research Institute; JYXM, JYXM2, and JYCQL2 were provided by the Beijing Yinong Vegetable Research Center; YueFeng, LiFeng2, LiFeng3, and ZaoQing8 were provided by the Vegetable Research Institute of Guangdong Academy of Agricultural Sciences; 15D036, 15D044, 15D088, 15D095, and YanBai were provided by the Chongqing Academy of Agricultural Sciences; ChuanCui13, ChuanLv11, ChuanLv15, ChuanCai19-1, ChuanCai19-2, and ChuanCai19-3 were provided by the Sichuan Academy of Agricultural Sciences. There were 40 plants in each variety, and we investigated whether there were more than three hollow fruits in each variety during the commercial fruit period.

### 4.2. Investigation on Hollow Trait of Cucumber Fruit

The hollow traits of the cross section in the middle segment of fruit at each stage of plant growth were investigated. The cross-sectional areas of the middle segment of all fruits were scanned with Deli altimeter (Version 15152, Deli Group, Ningbo, China). The total area and hollow area of each cross section were calculated using the Adobe Photoshop CS6 software. Finally, the percentage of hollow area was calculated with the statistical data of Microsoft Excel. The calculation formula was: Hollow area percentage = (hollow area/total area of cross section) × 100%.

### 4.3. Determination of Population Hollow Rate

H6 and H7 (20 plants each) were planted in the greenhouse of the Hunan Vegetable Research Institute, and managed with conventional water and fertilizer. After 40 days of pollination, the number of hollow fruits and the total number of fruits of the two materials were recorded. We calculated the population hollow rate according to the formula: Population hollow rate = (number of hollow fruits/total number of fruits) × 100%. From the spring of 2018 to the spring of 2019, the population hollow rate of the two materials was investigated three times.

### 4.4. Paraffin Sectioning

We collected the fruits of H6 and H7 plants at different stages (0 DAA, 2 DAA, 4 DAA, and 6 DAA). The middle segment of fruit was cut and fixed in FAA (formaldehyde-acetic acid-ethanol) fixative (50%) for 24 h. We took out the tissue from the fixed solution, trimmed the target tissue with a scalpel in the fume hood, and put the trimmed tissue and the corresponding label in the dehydration box. Then, it was dehydrated and dipped in wax with gradient alcohol in the dehydrator. The wax-soaked tissue was embedded in the embedding machine. The trimmed wax block was put into the paraffin slicer for slicing, with a thickness of 4 μm. The slices floated in the warm water at 40 °C of the spreader to flatten the tissue, then the tissue was placed on a glass slide and baked in an oven at 60 °C. After the water was dried and the wax was roasted, it was taken out and stored at room temperature for standby. The slices were put into xylene I for 20 min, xylene II for 20 min, absolute ethanol I for 5 min, absolute ethanol II for 5 min, 75% alcohol for 5 min, and rinsed with tap water. The slices were put into aniline blue dye solution for 5–10 min, washed with tap water, and dried in an oven at 60 °C. The slices were put into xylene for 5 min and sealed with neutral gum. Finally, the upright optical microscope was used for microscopic examination and image analysis data were collected. Three samples were collected in each period and each sample was repeated three times.

### 4.5. Whole Genome Resequencing

Twenty-five hollow fruit plant leaves and 25 non-hollow fruit plant leaves from the F_2_ separated population were selected to construct the mutant DNA pool and the wild-type DNA pool, named F_2_ hollow and F_2_ non-hollow, respectively. DNA was extracted using the CTAB method [53]. Two parental DNA pools (H6 and H7) and two DNA mixed pools (F_2_ hollow and F_2_ non-hollow) were obtained. DNA samples were randomly broken into 350 bp fragments with crusher (S220, Covaris, Woburn, MA, USA). The complete library was constructed by end repair, adding poly(A) tails, then adding sequencing connectors, followed by purification, PCR amplification, and other processes. After the library was constructed, we used Qubit 2.0 (Thermo Fisher Scientific, Waltham, MA, USA) for preliminary quantification. The library was diluted to 1 ng/μL and the insertion size of the library was detected using Agilent 2100 (Agilent Technologies, Inc., Santa Clara, CA, USA). The effective concentration of the library was accurately quantified by Q-PCR (effective library concentration >2 nm). After the qualified library was constructed, it was sequenced by Illumina NovaSeq (PE150, Illumina, Inc., San Diego, CA, USA).

### 4.6. SNP Calling and Filtering

The BWA (http://bio-bwa.sourceforge.net/; accessed on 29 January 2021) software (Version 0.7.12) was used to compare reads with 9930 cucumber reference genomes (http://cucurbitgenomics.org/, Version V2; accessed on 29 January 2021) [54]. The Samtools (http://www.htslib.org/; accessed on 29 January 2021) software (Version v0.1.18) was used to find genome-wide SNP sites [55]. The mutation results were detected using the GATK software (Version 3.2-2) [56]. The SNP filtering standard was that the base mass value was greater than or equal to 30, the mapping mass value was greater than or equal to 30, and the base depth was greater than or equal to 2 and less than or equal to 80 in two parents and two F_2_ mixed pools. The SNP index of the recessive pool minus the dominant pool was used for mapping.

### 4.7. SNP Genotyping by KASP

For the KASP genotyping, 346 F_2_ individual plants were used, including 259 plants that showed the hollow fruit phenotype and 87 plants that showed the non-hollow fruit phenotype. The SNP information of the target gene candidate region was detected on the LGC genomics typing platform at the Vegetable Center of the Beijing Academy of Agricultural and Forestry Sciences. The 100 bp DNA sequence upstream and downstream of each SNP site was intercepted as the KASP primer design template. A total of 3 primers (5′–3′ direction) were designed: Primer_Allele FAM, Primer_Allele HEX, and Primer_Common. The primer design information is shown in the Table S7.

### 4.8. Domain and Cis-Element Prediction

We used the InterProScan (http://www.ebi.ac.uk/interpro/; accessed on 26 February 2022) online analysis software to predict the protein domain [57]. The cis-acting elements of the promoter were analyzed by using the PlantCARE (http://bioinformatics.psb.ugent.be/webtools/plantcare/html/; accessed on 26 February 2022) online analysis software [58].

### 4.9. RNA Isolation and qRT-PCR

The middle segments in H6 and H7 fruit were collected at different stages (0 DAA, 8 DAA, and 40 DAA). Eight types of tissue samples were collected from H7, including sprout, flower, leaf, root, stem, tendril, and fruit. The samples of each material were collected repeatedly for three times. A TaKaRa MiniBEST Plant RNA Extraction Kit (Takara Bio Inc., Kusatsu, Japan) was used to extract the total RNA of the sample, and the test was carried out according to Protocol-II. The cDNA synthesis was performed using a TaKaRa PrimeScript RT reagent Kit with gDNA Eraser (Takara Bio Inc., Kusatsu, Japan). After reverse transcription reaction, TB Green Premix Ex Taq II (Tli RNaseH Plus) (Takara Bio Inc.) was used for RT-PCR detection. The primer was ALMT2-F: TTGGACGAGGATTGAATAGG; ALMT2-R: GAGCGACAGCATAATAGGT. *CsActin* was an internal reference gene. The 20.0 μL reaction system was as follows: TB Green Premix Ex Taq II (Tli RNaseH Plus) (2×), 10.0 μL; PCR Forward Primer (10 μM), 0.5 μL; PCR Reverse Primer (10 μM), 0.5 μL; ROX Reference Dye (50×), 0.4 μL; DNA template, 1.0 μL; ddH_2_O, 7.6 μL. The reaction was implemented in a fluorescence quantitative PCR instrument (ABI 7300, Thermo Fisher Scientific, USA). The relative gene expression levels were analyzed according to the 2^−∆∆Ct^ method. The internal normalization gene was *CsActin*.

### 4.10. Fluorescence In Situ Hybridization

The 0 DAA paraffin section of 2.4 was put into xylene for 15 min, xylene for 15 min, absolute ethanol for 5 min, and absolute ethanol for 5 min. The slices were dried under natural conditions and soaked in DEPC water. Protease K (20 μg/mL) was added dropwise and digested at 37 °C for 20 min. The slices were washed with pure water, and then washed with PBS, three times, for 5 min each time. Prehybridizing solution was added dropwise and incubated at 37 °C for 1 h. The prehybridization solution was poured out and the hybridization solution containing probe (*Csa1g630860* probe: 5′-CY3-AAGCCACAGUCGCCAUCGGUACUAUUUCCAG-CY3-3′) was added dropwise at a concentration of 6 ng/μL. For the hybridization reaction, the sample was placed in a 42 °C incubator for one night. The hybridization solution was washed off with 2 × SSC at 37 °C for 10 min, with 1 × SSC two times at 37 °C for 5 min each time, and then, washed with 0.5 × SSC at room temperature for 10 min. The slices were added with DAPI dye solution and incubated in the dark for 8 min. An anti-fluorescence quenching sealing agent was added to seal the slices after washing. The slices were observed under an upright fluorescence microscope (Eclipse CI, Nikon Corporation, Tokyo, Japan) and images were collected. The nuclei stained by DAPI were blue under UV excitation, and positive expression was red-light labeled with corresponding fluorescein (Cy3).

### 4.11. Subcellular Localization

The WoLF PSORT web server (https://wolfpsort.hgc.jp/; accessed on 11 March 2022) and CELLO version 2.5, subcellular localization predictor (http://cello.life.nctu.edu.tw/; accessed on 11 March 2022) were used to predict CsALMT2 subcellular localizations [59].

Primers for construction of the vector 1301-ALMT2-GFP were CZ-ALMT2-BglII-F: AACACGGGGGACTCTTGACCATGGAGATGGCTAATGAG and CZ-ALMT2-BglII-R: GCCCTTGCTCACCATATCTATAGTAACAACATGACAATG. The vector pCambia1301-JC-GFP was linearized by *Bgl*II enzyme digestion, and then reconstituted with the target gene fragment (the recombinant reaction kit is the ClonExpress-II One Step Cloning Kit of Vazyme Biotech Co., Ltd., Nanjing, China). The recombinant product was transformed into *E. coli* DH5α cells by heat shock method. Then, the vector sequencing results were analyzed. The constructed vector plasmid was transferred into *Agrobacterium* GV3101 and evenly plated on a kanamycin-containing plate. The single clones were picked and cultured in YEB liquid medium for 2 days in a shaker at 28 °C, and the cells were collected by centrifugation at 4000 rpm for 4 min. After removing the supernatant, the bacteria were resuspended in 10 mM MgCl_2_ containing 120 μM AS, and the OD_600_ was adjusted to about 0.6. The *A. tumefaciens* suspension was sucked up using a 1 mL syringe without a needle and pressed into the lower epidermis of tobacco leaves. Tobacco plants were cultured in low light for 2 days. After 3 days, the tobacco leaves in the injection area were taken to make slides, observed, and photographed under the laser confocal microscope. *A. tumefaciens* transformed with empty vector was used as a control.

## 5. Conclusions

Few studies have reported on hollow-related genes in cucumber fruit because of the environmental sensitivity of hollow trait and the unavailability of near-isogenic line materials. In this study, we used the non-hollow and hollow materials of cucumber fruit created by our research group and investigated them over multiple generations. The phenotypic analysis and cytological observations revealed that the hollow trait in cucumber fruit was formed in the early stage of fruit development with the opening of the carpel ventral zipper bi-cell layer. The genetic analysis showed that there may be a single dominant gene affected by the environment that controls the hollow trait in cucumber fruit. Through the BSA-seq and KASP genotyping analysis, the hollow gene of cucumber fruit was finely located, and the candidate gene was preliminarily determined as *CsALMT2*. Sequencing showed that the transversion G82A on exon 1 of *CsALMT2* resulted in an amino acid changing from alanine to threonine (A28T). Further, we used qRT-PCR and FISH to analyze the expression characteristics. The expression level of *CsALMT2* in H7 was significantly higher than that in H6, and showed tissue specificity that the ovule development zone inside the fruit had high expression. The *CsALMT2* gene may be localized to the plasma membrane. The results of this study should help to understand the molecular mechanism of the formation of hollow trait in cucumber fruit, and be benefical to develop molecular markers for seedling screening of high-quality cucumber varieties with non-hollow fruit.

## Data Availability

Not applicable.

## Supplementary Materials

The following supporting information can be downloaded at: https://www.mdpi.com/article/10.3390/ijms23116173/s1.
