# Supplementary material for: The Formation of Hollow Trait in Cucumber (Cucumis sativus L.) Fruit Is Controlled by CsALMT2
Source: Int J Mol Sci. 2022 May 31;23(11):6173. doi: 10.3390/ijms23116173 (PMC9181463; doi:10.3390/ijms23116173)
Supplement: Supplementary file 1 [file ijms-23-06173-s001.zip › ijms-1741281-supplementary.pdf]

Supplementary Material

The Formation of Fruit Hollow in Cucumber (*Cucumis sativus* L.) is Controlled by *CsALMT2*

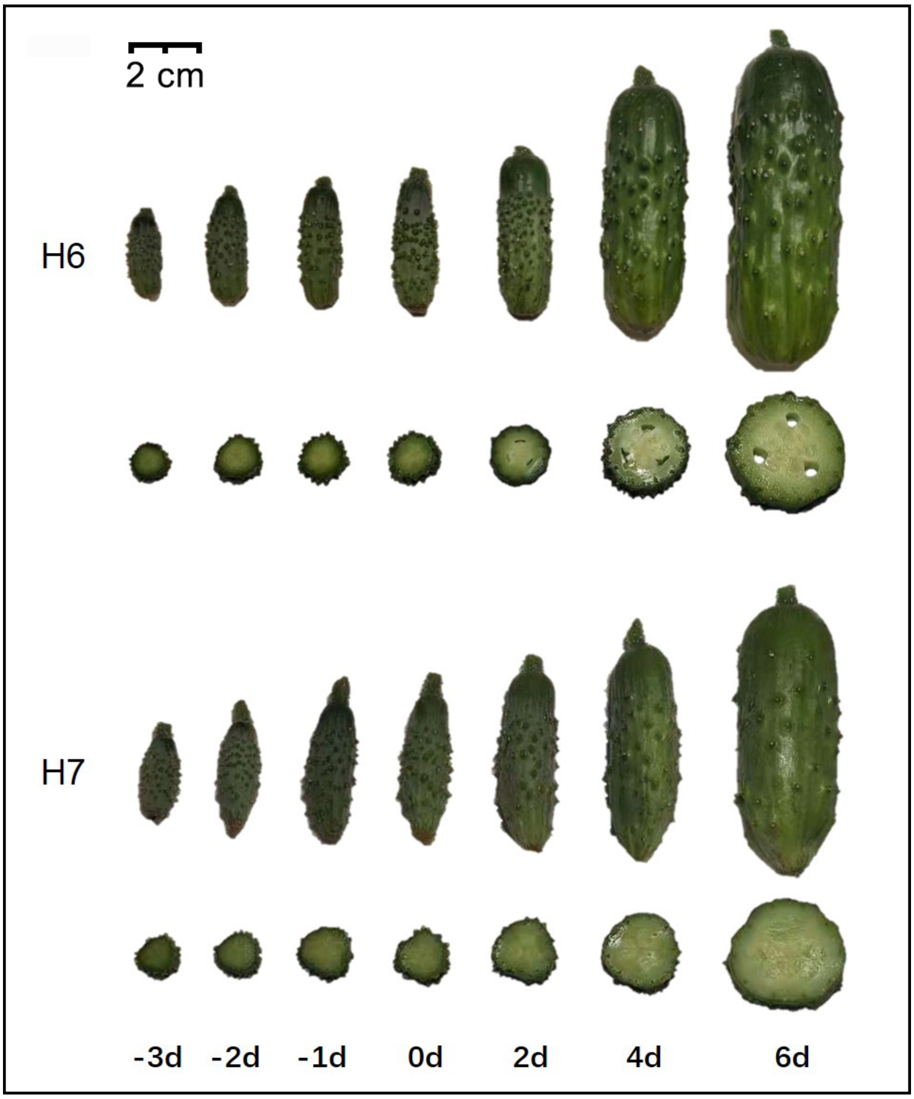

Figure S1. Hollow fruit trait of cucumber H6 and H7 from 3 days before flowering to 6 days after pollination. Scale bar = 1 cm.

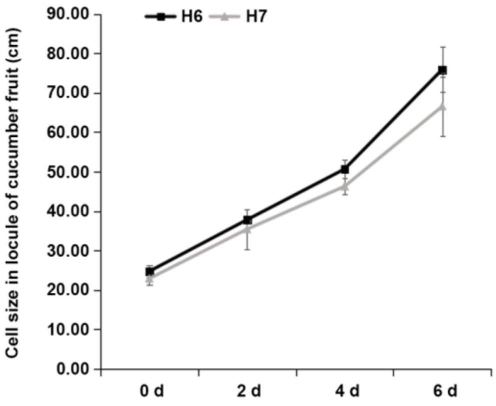

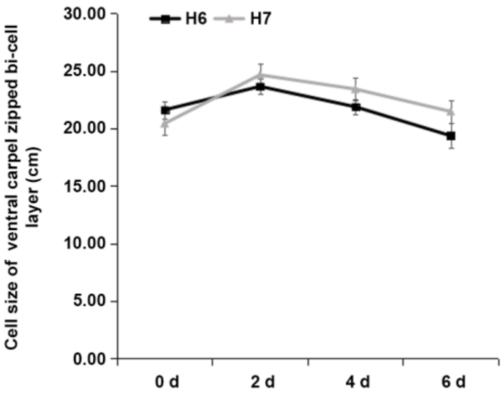

Figure S2. Cell size in locule and ventral carpel zipped bi-cell layer of cucumber fruit.

```
Benincasa_hispida          MEMANDKSEGVLARWGEGLEAKFSKLKAKVIELFRKTKKLAKDDPRRIVHSLKVGLALTL  60
Cucumis_melo               MEMSNEKSGGALARWSDGLKAKISKLKAKVIELFKKTKKLAKDDPRRIVHSLKVGLAITL  60
H7                         MEMANEKSGGLLASWSEGLKAKISKIMAKVIELFKKTKKLAKDDPRRVVHSLKVGLAITL  60
Cucumis_sativus_Gy14       MEMANEKSGGLLASWSEGLKAKISKIMAKVIELFKKTKKLAKDDPRRVVHSLKVGLAITL  60
Cucumis_sativus_PI183967   MEMANEKSGGLLASWSEGLKAKISKIMAKVIELFKKTKKLAKDDPRRVVHSLKVGLAITL  60
Cucumis_sativus_9930       MEMANEKSGGLLASWSEGLKAKISKIMAKVIELFKKTKKLAKDDPRRVVHSLKVGLAITL  60
H6                         MEMANEKSGGLLASWSEGLKAKISKIMTKVIELFKKTKKLAKDDPRRVVHSLKVGLAITL  60
                           ***:*:** * ** *.:**:**:**: :******:************:*********:**

Benincasa_hispida          VSLFYYFEPLYDGLGASAMWAILTVVVVFEFSVGATLGRGLNRVLATFFAAALGFGAHFL  120
Cucumis_melo               VSLFYYFEPLYDGLGASAMWAILTVVVVFEFSVGATFGRGLNRVLATFLAAALGFGVHFL  120
H7                         VSLFYYFEPLYDGLGASAMWAILTVVVVFEFSIGATLGRGLNRVLATFLAAALGFGAHFL  120
Cucumis_sativus_Gy14       VSLFYYFEPLYDGLGASAMWAILTVVVVFEFSIGATLGRGLNRVLATFLAAALGFGAHFL  120
Cucumis_sativus_PI183967   VSLFYYFEPLYDGLGASAMWAILTVVVVFEFSIGATLGRGLNRVLATFLAAALGFGAHFL  120
Cucumis_sativus_9930       VSLFYYFEPLYDGLGASAMWAILTVVVVFEFSIGATLGRGLNRVLATFLAAALGFGAHFL  120
H6                         VSLFYYFEPLYDGLGASAMWAILTVVVVFEFSIGATLGRGLNRVLATFLAAALGFGAHFL  120
                           ********************************:***:***********:*******.***

Benincasa_hispida          ADLAGDKAQPIMLSLSVFFL 140
Cucumis_melo               ADLAGDKAQPIMLSLSVFFL 140
H7                         ADLAGDTAQPIMLSLSVFFL 140
Cucumis_sativus_Gy14       ADLAGDTAQPIMLSLSVFFL 140
Cucumis_sativus_PI183967   ADLAGDTAQPIMLSLSVFFL 140
Cucumis_sativus_9930       ADLAGDTAQPIMLSLSVFFL 140
H6                         ADLAGDTAQPIMLSLSVFFL 140
                           ******.*************
```

Figure S3. Alignment of the multiple protein sequence of CsALMT2.

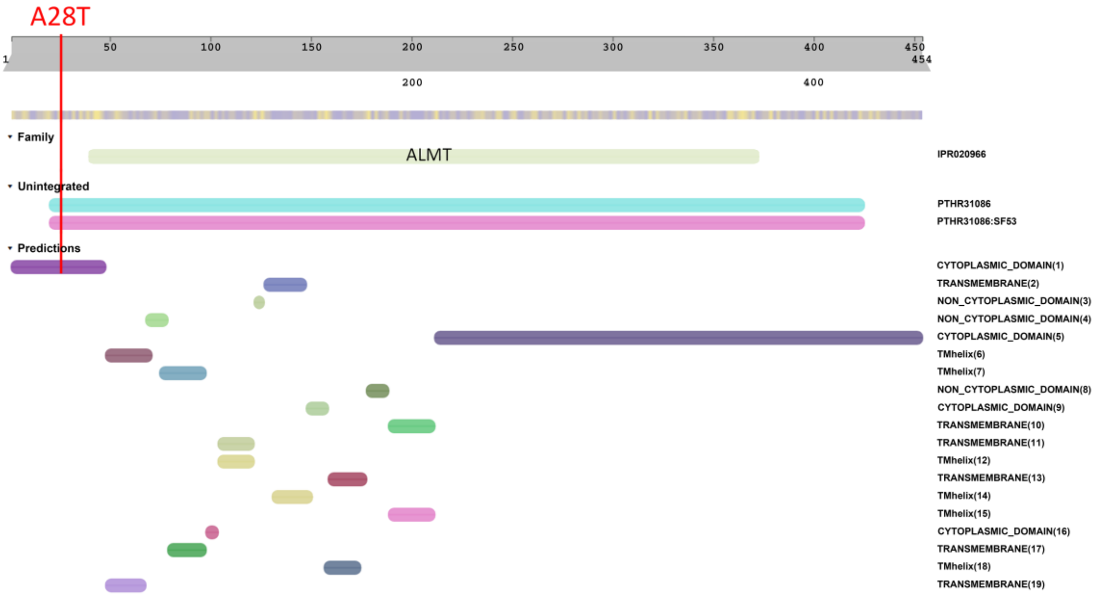

Figure S4. Domain analysis of CsALMT2.

Table S1. Investigation on hollow trait of cucumber varieties in different regions of China.

Classification	Variety number	Provinces	Hollow Character
North China type	ZhongNong38	Beijing	Non-hollow
	ZhongNong48	Beijing	Non-hollow
	ZhongNong106	Beijing	Non-hollow
	18C54	Beijing	Non-hollow
	18C56	Beijing	Non-hollow
	YD109	Jiangsu	Non-hollow
	YD110	Jiangsu	Non hollow
	JYXM	Beijing	Non-hollow
	JYXM2	Beijing	Non-hollow
	JYCQL2	Beijing	Non-hollow
	YueFeng	Guangdong	Non-hollow
	ChuanCui13	Sichuan	Non-hollow
South China type	HN1	Hunan	Hollow
	HN2	Hunan	Hollow
	HN3	Hunan	Hollow
	HN4	Hunan	Hollow
	LiFeng2	Guangdong	Hollow
	LiFeng3	Guangdong	Hollow
	ZaoQing8	Guangdong	Hollow
	15D036	Chongqing	Hollow
	15D044	Chongqing	Hollow
	15D088	Chongqing	Hollow
	15D095	Chongqing	Hollow
	YanBai	Chongqing	Hollow
	ChuanLv11	Sichuan	Non-hollow
	ChuanLv15	Sichuan	Hollow
	ChuanCai19-1	Sichuan	Non-hollow
	ChuanCai19-2	Sichuan	Hollow
	ChuanCai19-3	Sichuan	Hollow

Table S2. Percentage of average hollow area of P_1, P_2, F_1 and F_2 population plants.

Generation	Sample number	Percentage of average hollow area (%)	Range (%)	CV (%)
P_1	19	8.17±2.35a	4.16~13.11	28.72
P_2	20	0c	0.00~0.00	0
F_1	20	4.54±2.21b	1.33~10.74	48.67
F_2	345	4.95±3.92b	0.00~19.54	79.17

Note: Values followed by different letters (a-c) : Significantly different (p < 0.01).

Table S3. Frequency distribution of hollow area percentage.

Generation	Hollow area percentage (%)											Sample number	Sample average (%)	Standard deviation (%)
	0.0	0.1–2.0	2.1–4.0	4.1–6.0	6.1–8.0	8.1–10.0	10.1–12.0	12.1–14.0	14.1–16.0	16.1–18.0	18.1–20.0			
P_1	-	-	-	3	7	6	1	2	-	-	-	19	8.17	2.35
P_2	20	-	-	-	-	-	-	-	-	-	-	20	0	0
F_1	-	2	6	8	3	-	1	-	-	-	-	20	4.54	2.21
F_2	7	91	65	60	49	36	15	13	6	1	2	345	4.95	3.92

Table S4. Raw sequencing data statistics.

Sequencing data ID	Number of raw reads	Number of raw bases	GC%	Bases Q30%
F_2 hollow	44,635,824 bp	6,695,373,600 bp	38.91%	91.79%
F_2 non-hollow	40,274,958 bp	6,041,243,700 bp	39.19%	91.86%
H6	36,593,864 bp	5,489,079,600 bp	39.59%	91.40%
H7	38,006,642 bp	5,700,996,300 bp	39.46%	90.29%

Table S5. Comparison data statistics.

Sequencing data ID	Genome length	Number of bases compared	Coverage	Average depth
F_2 hollow	196,481,733 bp	190,317,945 bp	97.00%	17.68
F_2 non-hollow	196,481,733 bp	190,672,050 bp	97.00%	23.14
H6	196,481,733 bp	190,568,736 bp	97.00%	21.46
H7	196,481,733 bp	190,435,487 bp	97.00%	19.12

Table S6. Plant cis-acting regulatory elements prediction of *CsALMT2*.

Promoter element	Sequence	Function	Organism
ABRE	ACGTG	cis-acting element involved in the abscisic acid responsiveness	*Arabidopsis thaliana*
ACE	CTAACGTATT	cis-acting element involved in light responsiveness	*Petroselinum crispum*
ARE	AAACCA	cis-acting regulatory element essential for the anaerobic induction	*Zea mays*
AT-rich sequence	TAAAATACT	element for maximal elicitor-mediated activation (2copies)	*Pisum sativum*
G-box	TACGTG	cis-acting regulatory element involved in light responsiveness	*Arabidopsis thaliana*
GCN4-motif	TGAGTCA	cis-regulatory element involved in endosperm expression	*Oryza sativa*
MBS	CAACTG	MYB binding site involved in drought-inducibility	*Arabidopsis thaliana*
P-box	CCTTTTG	gibberellin-responsive element	*Oryza sativa*
TC-rich repeats	GTTTTCTTAC	cis-acting element involved in defense and stress responsiveness	*Nicotiana tabacum*
TCA-element	CCATCTTTTT	cis-acting element involved in salicylic acid responsiveness	*Nicotiana tabacum*
TGA-element	AACGAC	auxin-responsive element	*Brassica oleracea*

Table S7. KASP primer information.

Cucumber-SNP ID	Primer name	Primer sequence
CHM1_19454233	Primer_AlleleX	GAAGGTGACCAAGTTCATGCTATACCTCGTTCAGTTCTTCTGTGTG
	Primer_AlleleY	GAAGGTCGGAGTCAACGGATTTAATACCTCGTTCAGTTCTTCTGTGTA
	Primer_Common	GAAGAGCAGATAAACATACCCCTTCAAAA
CHM1_19830655	Primer_AlleleX	GAAGGTGACCAAGTTCATGCTCTGCGTTACAGGAAGACTGTCAG
	Primer_AlleleY	GAAGGTCGGAGTCAACGGATTACTGCGTTACAGGAAGACTGTCAA
	Primer_Common	CGGAGGGGATGGAAATGATAGAGTT
CHM1_20065780	Primer_AlleleX	GAAGGTGACCAAGTTCATGCTCATCGCTATCAGTGGCCAAGATAAT
	Primer_AlleleY	GAAGGTCGGAGTCAACGGATTATCGCTATCAGTGGCCAAGATAAC
	Primer_Common	GTTAGATAATCATTTTGCTTAACCTACAAGGTTT
CHM1_21642977	Primer_AlleleX	GAAGGTGACCAAGTTCATGCTAGGAATGCCAGCTAAAATCATTCTTGA
	Primer_AlleleY	GAAGGTCGGAGTCAACGGATTGGAATGCCAGCTAAAATCATTCTTGC
	Primer_Common	TAACAATGGTGAAATGGATGACTTTCTAGTT
CHM1_21892895	Primer_AlleleX	GAAGGTGACCAAGTTCATGCTCTTGTACATTTTCAATCATTGGGATTGC
	Primer_AlleleY	GAAGGTCGGAGTCAACGGATTCTTGTACATTTTCAATCATTGGGATTGT
	Primer_Common	CATCGCCGGTGGTGACTTGACTT
CHM1_25854734	Primer_AlleleX	GAAGGTGACCAAGTTCATGCTGGAAGTACCAATGCAAGTATTCCCT
	Primer_AlleleY	GAAGGTCGGAGTCAACGGATTGAAGTACCAATGCAAGTATTCCCC
	Primer_Common	CTCCTAGCCCTAGTGATCCTAATCTT
CHM1_28330443	Primer_AlleleX	GAAGGTGACCAAGTTCATGCTCTATGTACTGATGAAGGAGTATGAGAAT
	Primer_AlleleY	GAAGGTCGGAGTCAACGGATTTATGTACTGATGAAGGAGTATGAGAAG
	Primer_Common	GCAAGAAACTGTAGTCTCAATGAAGCAA

	Primer_AlleleX	GAAGGTGACCAAGTTCATGCTCTACCCAAAGCTCCGATTCCGA
CHM1_28340789	Primer_AlleleY	GAAGGTCGGAGTCAACGGATTTACCCAAAGCTCCGATTCCGG
	Primer_Common	TACCGTTTCCTTCGGCCGCCTT
	Primer_AlleleX	GAAGGTGACCAAGTTCATGCTCTAATCTTTTCTAAAGGGATCTAGTTTGTTT
CHM1_28663072	Primer_AlleleY	GAAGGTCGGAGTCAACGGATTCTAATCTTTTCTAAAGGGATCTAGTTTGTTA
	Primer_Common	GTCAAACATGAGTTGGAAACCTCTTGTATA
	Primer_AlleleX	GAAGGTGACCAAGTTCATGCTCTACATCTAATCCTTATTCTTGAGCAGAA
CHM1_28681211	Primer_AlleleY	GAAGGTCGGAGTCAACGGATTACATCTAATCCTTATTCTTGAGCAGAG
	Primer_Common	CATCACATTGTTGACATGAATCCAACTGTA
	Primer_AlleleX	GAAGGTGACCAAGTTCATGCTTTCTTTTCAATAAGCGACATTATTTCTCGA
Chr1_21902047	Primer_AlleleY	GAAGGTCGGAGTCAACGGATTTTCTTTTCAATAAGCGACATTATTTCTCGT
	Primer_Common	ATATGGAAAATTGAGAGGTAGAAAATGGAAATAA
	Primer_AlleleX	GAAGGTGACCAAGTTCATGCTCCTTAAAACAGTCTCTCTAGCCACTA
Chr1_22288154	Primer_AlleleY	GAAGGTCGGAGTCAACGGATTCCTTAAAACAGTCTCTCTAGCCACTT
	Primer_Common	CACCCTTTCAAGAGAAAAAGGGCCTTT
	Primer_AlleleX	GAAGGTGACCAAGTTCATGCTTGGGATTAAGATTAGGATTAGTTTACTTTTG
Chr1_23662100	Primer_AlleleY	GAAGGTCGGAGTCAACGGATTATTGGGATTAAGATTAGGATTAGTTTACTTTTA
	Primer_Common	CTACTACAGTTAATTTTTTAATAAGAGAAACTGAA
	Primer_AlleleX	GAAGGTGACCAAGTTCATGCTAAACATACATGAACGTTTCAAACGTCAC
Chr1_24702649	Primer_AlleleY	GAAGGTCGGAGTCAACGGATTATAAACATACATGAACGTTTCAAACGTCAT
	Primer_Common	AACTAAATTATTGTTTAAAGTTGCGACTTGAAATT
	Primer_AlleleX	GAAGGTGACCAAGTTCATGCTGAGCTAAGGTGGGACGACAAAAC
Chr1_24906295	Primer_AlleleY	GAAGGTCGGAGTCAACGGATTTGAGCTAAGGTGGGACGACAAAAT
	Primer_Common	TTTGGCCGCCGTCACAAGCTAAAAT

	Primer_AlleleX	GAAGGTGACCAAGTTCATGCTGCGTTTAGGGTTTTGGTTCTGTTTTG
Chr1_25118463	Primer_AlleleY	GAAGGTCGGAGTCAACGGATTGCGTTTAGGGTTTTGGTTCTGTTTTC
	Primer_Common	TGCAATTCCAAGAATCTTCAGTCAGCTT
	Primer_AlleleX	GAAGGTGACCAAGTTCATGCTCTATCTTTCCTCCCATTTTCCTTCTTT
Chr1_25185078	Primer_AlleleY	GAAGGTCGGAGTCAACGGATTTATCTTTCCTCCCATTTTCCTTCTTG
	Primer_Common	GGGAGAACCTGAGTTGAGATTGAATATAT
	Primer_AlleleX	GAAGGTGACCAAGTTCATGCTATCAACCAACAACATCAGCCTCAAC
Chr1_25203154	Primer_AlleleY	GAAGGTCGGAGTCAACGGATTGATCAACCAACAACATCAGCCTCAAT
	Primer_Common	GTTTTGGTCTTTACTTCTTATGATTGTAGTAAATT
	Primer_AlleleX	GAAGGTGACCAAGTTCATGCTGTCTAAATCGCCAAAATTGAACACTTAAAT
Chr1_25430140	Primer_AlleleY	GAAGGTCGGAGTCAACGGATTTCTAAATCGCCAAAATTGAACACTTAAAC
	Primer_Common	CCCACTTCTAATTTGAGTATAAGAAGTAAAATTT
	Primer_AlleleX	GAAGGTGACCAAGTTCATGCTCAACCAACTGACTTCAGTTTGGTCA
Chr1_25510362	Primer_AlleleY	GAAGGTCGGAGTCAACGGATTAACCAACTGACTTCAGTTTGGTCG
	Primer_Common	TTAGGGGTGAGCATGATAGGTTGAAAAA

Table S8. Amino acid sequence for construction phylogenetic tree of ALMT2.

Name	Amino Acid Sequence
CsALMT2	MEMANEKSGGLLASWSEGLKAKISKIMAKVIELFKKTKKLAKDDPRRVVHSLKVGLAITLVSLFYYFEPLYDGLGASAMWAI<br>LTVVVVFEFSIGATLGRGLNRVLATFLAAALGFGAHFLADLAGDTAQPIMLSLSVFFLAAITTFVRFFPRIKARYDYGFLIFILT<br>FCLVSVSGYREDEILKVAYRRALTILIGTFIAILICILICPVWAGDDLHSLVSNNIEQLANFFQGFGVEYSNEWKEDEGIVEGFKS<br>VLTSRQTEESLVNFARWEPGHGTFKFRHPWKQYRKIGSLTRQCAYRLESLNTYLLAESQTPLHIRDQLKESCSKMSTESGKAL<br>KDLASSIRTMTLPRLPNPHIEKSKAAAKDLKAALKIRPCNSSIDLLEIVPMATVASLLIDSISCIEKIAESVGELASLANFKRFEV<br>EKSASLKFQQEQQQKLATPAIVSGHCHVVTID
CmALMT2	MEMSNEKSGGALARWSDGLKAKISKLKAKVIELFKKTKKLAKDDPRRIVHSLKVGLAITLVSLFYYFEPLYDGLGASAMWAI<br>LTVVVVFEFSVGATFGRGLNRVLATFLAAALGFGVHFLADLAGDKAQPIMLSLSVFFLAAITTFVRFFPRIKARYDYGFLIFILT<br>FCLVSVSGYREDEILKVAYRRALTILIGTFIAILICILICPVWAGDDLHSLVSNNIEQLANFFQGFGVEYSNEWKEDERIVEGFKS<br>VLSSRQTEESLVNFARWEPPHGTFKFRHPWKQYSRIGSLTRQCAYRLESLNTYLLAESQTPLHIRDQLKESCTKMSTESGKAL<br>KDLASSIRTMTLPRLPNPHIEKSKAAAKDLKAALKIRPCNSSIDLLEIVPMATVASLLIDSISCIEKIAESVGELASLANFKCLEV<br>GKSASLKFQQEQQQKLATPAIVSGHCHVVTVD
BhALMT2	MEMANDKSEGVLARWGEGLEAKFSKLKAKVIELFRKTKKLAKDDPRRIVHSLKVGLALTLVSLFYYFEPLYDGLGASAMWA<br>ILTVVVVFEFSVGATLGRGLNRVLATFFAAALGFGAHFLADLAGDKAQPIMLSLSVFFLATITTFVRFFPRLKARYDYGFLIFIL<br>TFCLVSVSGYREDEILNVAYRRALTILIGTFIAILICICICPVWAGDDLHSLVSNNIELLANFLEGFGGQYSNEWKENEVVEECK<br>SVLTSRQTEESLVNFARWEPGHGTFKFRHPWKQYRKIGSLTRQCAYRLESLNTYLVTECQTPLHIRDQLKESCSKMSIESSKAL<br>KVLASAIRTMTRPTFPNPHIERSKAVAKDLKAALKIRPCDSGMDLLEIVPMATVASLLIDSISCIEKIAESVGELASLANFKCVE<br>VGKSASMKLEQHQEQKLPTPAIVNGHCHVVTID
McALMT2	MEMADHKSEGVSSARWAEGLRAKSSRLTVKVGELARKTKKLAKDDPRRIAHSLKVALAITLVSLFYYFEPLYDGFGASAMW<br>AILTVVVVFEFSVGATLGRGLNRVLATFSAAALGFGAHFLADLAADKGEPIILAFFVFLLAATTTFVRFFPRIKARYDYGFLIFI<br>LTFCLVSVSGYRDDEILQVAYTRALTILIGTFIAILICIFICPVWAGEDLHSLVSNNVELLGNFLQGFGARYSDEWKGDKQVEGC<br>KSVLTSRQTEESLVNFARWEPGHGSFKFRHPWKQYRKIGSLTRQCAYRLESLNGYLTTETQIPLHIRDQLKDSCSKMSIESGKA<br>LKDLASAIRTMTRPTFPNPHVEKSKAAAKNLKVALKIGPCNDGIDLLEIVPAATVASLLIDSISCIEKIAESVGELASMAQFKK<br>CVEAGKLAGLELEQDQQQNLPTPAIVNGHCHVV

CcALMT2	ESIKALLGKMRTKVTEFPGKIKKLGQDDPRRIVHSLKVGLAITLVSLFYYFDPLYVGFGSSAMWAVLTVVVVFEFSVGATLGK<br>GLNRGLATFLAGSLGFGAHHLATLPGEKFQPILLGFFVFLLATTVSFIRFFPRMKARYDYGLVIFILTFCLISVSGYRDEEVLELA<br>HKRVSTILIGGFTALFVCIFICPVWAGDDLHNLAANNIEKLANFLEEFGDEYFKESKDRESNKASLQGYKSVLNSKQNEESLV<br>NFARWEPRHGRFRFRHPWKQYQKIGSLTRHCAYRVEALNGYLNSDVKASLETRGKIKEACMKLSCESGKALKELASALRT<br>MTQPPSQCPHIAKSKNAANSLKSLLKTGLCKDIDLQEIVAVATVASLLLDVLSCTERISESVHEIASLARFKSLEDDKVADGAK<br>INQKGQVNNKPCSNSIEQPNHHVITID
XsALMT2	VADQKEAGMIFRGWGWLKAFPGKIISKVVEFARKTKKLGQDDPRRIIHSLKVGLAITLVSMFYYFEPLYDGFGVSAMWAVLT<br>VVVVFEFSVGATLSKGFNRGLATFLAGTLGFGAHHLAILSGEKAEPILLGLFVFLLAAMMTFVRFFPRMKAKYDYGVLVFILT<br>FCLISVSGYRDDEVLEMAHKRVSTILLGGFAALLVCIVICPVWAGNDLHNLVANNIEQLASFLEGFGPEYFNTSEDVESNNTL<br>LEGYKSVLHSKQTEESLANFARWEPCHGQFRFGHPWKHYLKIGSLTRKCAYRIEALHGYLNTKNQTSQEIRSKIQDESIKVSS<br>ESVKALKELAMAIETMITPSAATLHITKSKIAAKNLKLMLNADLCKETDLLEVIPAVTMASLLVDVVSCSEKIAESVHELASL<br>AKFKSVKPEVAARKPSLQKMQSYSSVNVSQSHHVITVE
CoALMT2	ESIKALVGKMRTKVTEFPGKIKKLGQDDPRRIVHSLKVGLAITLVSLFYYFDPLYVGFGSSAMWAVLTVVVVFEFSVGATLGK<br>GLNRGLATFLAGSLGFGAHHLATLPGEKFQPILLGFFVFLLATTVSFIRFFPRMKARYDYGLVIFILTFCLISVSGYRDEEVLELA<br>HKRVSTILIGGFTALFVCIFICPVWAGDDLHNLAANNIEKLANFLEEFGDEYFKESGDRESNKASLQGYKSVLNSKQNEESLV<br>NFARWEPRHGRFRFRHPWKQYQKIGSLTRHCAYRVEALNGYLNSDVKASVETRGKIKEACMKLSSESGKALNELALAVRT<br>MTQPPSECPHIAKSKNAANSLKSLLKTGLCKDIDLQEIVAVATVASLLLDVLSCTEKISESVHELASLARFKGVEDDQVADGE<br>KKLNQKGEVNNKSCSNSIEQPNHHVITID
PdALMT2	QNNGGCFSRGCGWIKSLPERSKAKIVECARKIKKLGQDDPRRVNHSVKVGLAITLVSLFYYFEPLYDGFGDSAMWAVMTVV<br>VVFEFSVGATLGRGLNRGLATFLAGALGFGAHRLATLSGEKGEPMLLGLFVFLLATTVTFVRFFPRMKARYDYGLLIFILTFC<br>LISVSGYRDDEVLDMAHKRVSTILIGGLTAVLVCICICPVWAGDDLHNLAATNIEKLGIFLEHFGVEFFRKPGEGESINKASLQ<br>GYKSVLNSKNMEESLVNFARWEPGHGQFKFRHPWKHYLKFGSLTRQCAYRVEALNGYLNSDIKTPPEIQGMIQDSCTKMSS<br>ELGKALKELALAIKRMTPPSSASSHLVKSKNAAKNLKFLLYSDLCSGINLLEVVPAVTVTSLLFEVISCTEKIAEAIHELASLAQ<br>FENVEQERPKLPEQGEMQQGANMDVHHHVVTID
CiALMT2	VSGWGRLKALPGRLGTKIVEAAMKIKKLGQDDPRRIIHSLKVGLAITLVSLIYYVESLYEGFGGSAMWAILTVIVVLEFSVGAT<br>LGRGLNRGLATFIAGALGFGAHYLASRSGDKVEPMLLGLFVFLIAGLVTFIRFFPQMKARYDYGLLIFMLTFCLVSISGYRDED<br>LLDVAHRRVSTILIGSFTSVFVCICICPVWAGDDLHNLVASNIENLGSFLQGFGIEYFKISGEFGESKTAVMQRYKTVLNSKQSE<br>SLANFARWEPGHGQFRFSHPWKQYLKIGSLTRQCAYRIDALNGYLNSEIRTPPEFLSKIQATCTMMSMESGKALTELASVIKT

	MTPPSSAKPHIAKSENAAKGLKSLLQAGLCKDIDLLEVLPAATVASLLVDVVSSTEKITESINELATLARYESIEHIIVDEKEAA<br>ESLHLQPIAPCSNTSGEHHVITID
ArALMT2	SHAEAGPFTRAWHWLKALPEKLWSKFVSVATKAKKLGQDDPRRIVHSLKVGLAITLVSLFYYFEPLYDGLGVSAMWAVLTV<br>VVVFEFSVGATLGRGVNRGLATLLAGALGFGAHRLASFSGNICEPILLGLFVFLLAAVVTYIRFFPRMKARYDYGLLIFILTFCL<br>ISVSGYRDDEVLDMAQKRLSTILIGGATALFVCVFICPVWAGDDLHNLAAINMEKLGSFLEGFGNEYFKTPSQGQSMEDKEI<br>LQGYRSVLNSKTTEESLANFAKWEPRHGRFRYRHPWQQYLRIGSLTRQCAYRIEALNGYLSSELKASQEIRAKIEDPCKKMSK<br>DCSCALKELAQAVKTMTKPSAANCHIANSKIAAKSLKSMLKTGLWQDTDLLEIVPAATVASLLIDIVSSTEEIAESVNELASL<br>ANFRSVDNTEVAKGESKLSCSSSIKGPHHIITI
ZjALMT2	SGGRLGFGRVKALFEKLKNWVVEIAMKTKKLGEDDPRRIVHSLKVGLAVSLVSLFYNFDFVYDGFGSSAMWAVLTVVVVFE<br>FSVGATLGRGLNRGLATFLAGALGFGAHHLANLSGDKVQPILLTLFVFLLAACVTFIRFFPRMKARYDYGLLIFILTFCMISVS<br>GYRDEEILEMAHKRVSTILIGGLTALSVCILICPVWAGDDLHNSVANNIEKLGNFLEGFGNVYFKKSEDGESNKAVLQGYKS<br>VLNLKQSEESQVNFARWEPRHGKFRFRHPWKHYLTVGTLTRQCAYRIDALNGYIQSEIQAPLEIGSEMEEACMKMSSESGK<br>ALKELAATMRTMTLPCSGGGAAPHIAKSRLAAKSLKSLLKLKTGLMLEDNTSDLLNLVPAATVASLLVDVVCCTEKIDKAIE<br>ELASLAKFKSSEPTMEDHKPKLNRQMAFLQQRSTSFGPHHVVTI
MnALMT2	LKNLAENVRAKVSKVANNVKKLGKDDPRRIIHSLKVGVSLSLVSIFYYYQPLYDNFGVSAMWAVMTVVVVFEYTVGATLGK<br>GLNRGLATLLAGALGVGAHHLASLSGDIGEPILLGFFVFLQAATSTFIRFFPKVKARYDYGLLIFILTFSLISVSGFRDDEILEMA<br>HKRLSTILIGGSACVVISILVCPVWAGEDLHNLIALNLEKLGNFLEGFGDEYFNNTAMNEESKDDKAYLQGYKSVLNSKSSEE<br>SLANFARWEPGHGRFKFRHPWKQYLKVGTLTRQCAYRIEALHGRLSADKIQTSPEIREQIQEACMKVSSESGKALKELATSV<br>KKMTKPSSANPHLASSKVAAKSLRSLLKSGSWDNIDLLEVIPSVTVASILLDVVNCTEKIAESVNELASLAHFK
HsALMT2	KIRRKVAEFPEKIKKVAQDDPRRVVHSFKFGLAITLVSLFYYFDPLYVGFGNSAMWAILTVVVVFEFSVGATLVKGLNRGLATF<br>LAGALGFGVHHLATLPGDIVRPILLGIFVFILATTVSFIRFFPRMKARYDYGLLIFILTFCLISVSGYRDEEVLQMAHKRVSTILIG<br>ALAALLVCIFICPVWAGDDLHNLAANNLENLATFLQGFGDEYFTKSSEMESNKASLQGYKSILNSKQVEESWVNSARWEPR<br>HGRFKFRHPWKQYLMVASLTRQCAYRVEALNGCLDSDVQASPETCGKFQETCTEISSESGKAMKELASAIKTMSAPSSHRA<br>HIAKAKDATEKLNSLLKTRLCEDIDVVEILAVATVASLLHDVLACTEKISESVHELATLAHFKNEKDGKQELDKQKQVQKTS<br>NSIDLNHHVITVE
GhALMT2	GGSSNSWLALVDSIVKIRSKVAEFPGKMKKVAEDDPRRIVHSFKVGLAITLVSLFYYFDHLYVGFGSSAMWAVLTVIVVIEFSV<br>GATLGKGLNRGLATFLAGTLGFGAHHLANLPGRELQPILLGIFVFILAATVSFIRFFPRMKVRYDYGLLIFMLTFCLISVSGYR

	DEEVLEMAHKRVSTILIGGFTAIFVCIFICPVWAGEDLHNLAANNLEKLASFLEGFGDEYFRKSSEPESNKASLQAYKSILNSK<br>QSEESLVNFARWEPPHGPFRFRHPWKQYLKIGSLTRQCAYRIEALNGCLNSDVQASPETCRNFREAFIKASSETGKAVKGLAS<br>AMRTMTLPTGDCPQTAKSKNAADNLKSLLRTGLCEGIDIVEILEVATVASLLLDILACTEKISESIHELASLAHFKRLEPDEKD<br>GNPDLDKQKQVWQSSNSIASNHHHHIITIE
MiALMT2	EKAGFCAGAWLLLKGLPEKLKHKVAELASKAIKLGKDDPRRVVHSLKVGLALNVVSLLYYLRPLYNDFGVSATWAIMTVVF<br>VFEYSVGATLGKGLNRGFATFIGGALSVGAHQIACLSGEKAEPIMLGLFVFLQAAASTFLRFFPKIKARYDYALLIFILTFSMIS<br>VSGFRDDEIIDLARRRLLTILIGGFVCVIICILICPVWAGQDLHNLIATNIDKLGNFLEGFGNEYFNKEGETEDDKSFLQQYKSV<br>LTSKSTEESLTNSAKWEPGHGQFQFRHPWNLYLKIGTLTRQCAYRIDALNGYLNAEIQTSSEMRYKIEEVYREMSLESGKALK<br>ELALAIKTMSQPSAATKTHIENSKSAAKSLNSLFKSNIWGDTDLQAAVPVAAVASLLIDVVACTEKIAQSVYELASMANFKSI<br>EVTLSPEKSQIRQQDNAKSLAKVDDCPYVCVTVN
SpALMT2	KIFANIVGIAKQFKKNAKDDPRRVIHSLKVGLSLTLVSLFYYFQPLYNGFGVSTMWAIMTVVVVFEFSVGATLGKGLNRGMA<br>TLLAGALGVGAHYLASASGKVVEPILLGLFVFLQAFALTFIRFFPQVKARYDYGMLIFILTFCLVSISGFRVDEIVDLAHKRISTI<br>LMGASVCVIVSIFVCPVWAGEDLHKLVAKNMEKIGNFLEGFGDEIFKSSEDITESKVAKPSLIEYKSVLNSKNTEETLANFAK<br>WEPGHGQFKYRHPWKQYLKIGGLIRQCACRIDALNGYINSEIKAPEEIKEMIKETSMKMSIECGKALKELSKSMRKMNLPIS<br>ADEHVTNAKNSAKKLNSLLKSRINNWEEMNLLQVIPLTTIASILRDIVTCVEEIGQGVNELASLANFK
SlALMT2	KIFANIVGIAKQFKKNAKDDPRRVIHSLKVGLSLTLVSLFYYFQPLYNGFGVSTMWAIMTVVVVFEFSVGATLGKGLNRGMA<br>TLLAGALGVGAHYLASASGKVVEPILLGLFVFLQAFALTFIRFFPQVKARYDYGMLIFILTFCLVSISGFRVDEIVDMAHKRIST<br>ILMGASVCIIVSIFVCPVWAGEDLHKLVAKNMEKIGNFLEGFGDEIFKSSEDITESKVAKPSLIEYKSVLNSKNTEETLANFAK<br>WEPGHGQFKYRHPWKQYLKIGGLIRQCACRIDALNGYINSEIKAPEEIKEMIKETSMRMSIECGKALKELSKSMRKMNLPIS<br>ADEHVTNAKNSAKKLNSLLKSRINNWEEINLLQVIPLTTIASILRDIVTCVEEIGQGVNELASLANFK
CaALMT2	TNGLLWFKSLYEKLFAKIIGIAKQFKKIGKDDPRRVIHSLKVGVALTLVSLFYYFQPLYNGFGVSTMWAIMTVVVVFEFSVGAT<br>LGKGLNRGMATLLAGALGVGAHYLASASGKVVEPILLALFVFLQAFATTFIRFFPQVKARYDYGMLIFILTFCLVSISGFRVDE<br>IVDMAHKRLSTILIGASVCVIISIFVCPVWAGEDLHKLVANNMEKLGKFLEGFGDEIFKSAEVEESKDAKKSLIEYKSVLNSKN<br>AEETLANFARWEPGHGQFKYRHPWRQYLKIGGLIRQCACRIDALNGYINSEIKAPEEIKEKIKETSKEMSIECGKALKELSNS<br>VRKMNLPISADEHITNAKKSAKNLNSLLKSGINNWEEINLLQVIPLATIASILTDIVICVDEIGQGVNELATLAHFKK
AtALMT2	MEKVREIVREGRRVGKEDPRRVVHAFKVGLALALVSSFYYYQPLYDNFGVNAMWAVMTVVVVFEFSVGATLGKGLNRAVA<br>TLVAGGLGIGAHHLASLSGPTVEPILLAIFVFVLAALSTFVRFFPRVKARYDYGVLIFILTFALISVSGFREDEILDLAHKRLSTVI

	MGGVSCVLISIFVCPVWAGQDLHSLLASNFDTLSHFLQEFGDEYFEATEDGDIKEVEKRRRNLERYKSVLNSKSNEEALANF<br>AKWEPRHGQFRFRHPWRQYLAVGALLRQSAYRIDALNSNINSDMQIPMDIKKKIEEPLRRMSSESGKSMKEVSISLKNMTIS<br>SSFDIHVVNSQSACKTLSTLLKSGILNDVEPLQMISLMTTVSLLIDIVNLTEKISESVHELASAA
AtALMT7	MEKVREIVREGRRVAKEDPRRVVHSFKVGLVLALVSSFYYYQPLYDSFGVNAMWAVMTVVVVFEFSVGATLGKGLNRVAAT<br>LFAGGLGIGAHHLASMSGPTGEPILLAVFVFVQAALSTFVRFFPRVKARYDYSLLIFILTFALISVSGFREEQVVKLTHKRISTVII<br>GGLSCVIISIFVCPVWAGQDLHSLIASNFEKLSFFLLGNSFHYVSSDLNSITLLRKIKSWRLADFGDKYCEVVENDGAKEVDKR<br>KKDFDNYKSVLNSKSNEESLANFAKWEPGHGQFRFRHPWKQYLAVGELIRQCAYRIHALNSYLNADNQVSVDIKKKLGEP<br>LRRMSLESGKAMKEMSISLKKMTKPSSSDLHVQNAKSACKSLTNLLNSGILKEVEPLELVSLLTAISLLIDIINLTEKILESLHEL<br>ATAAKFK
AtALMT8	AQEKKAGFFQRLQDFPSKLKDDVTKRVKNVQKFAKDDPRRIIHSMKVGVALTLVSLLYYVRPLYISFGVTGMWAILTVVVVF<br>EFTVGGTLSKGLNRGFATLIAGALGVGAVHLARFFGHQGEPIVLGILVFSLGAAATFSRFFPRIKQRYDYGALIFILTFSFVAISG<br>YRTDEILIMAYQRLSTILIGGTICILVSIFICPVWAGEDLHKMIANNINKLAKYLEGFEGEYFQPEKISKETSSCVREYKSILTSKST<br>EDSLANLARWEPGHGRFRLRHPWKKYLKIAGLVRQCAVHLEILNGYVLSNDKAPQEFESKIQEPITTMSREVGEALKAIAKS<br>IKTMRNDSACVNAHIDNSKKAIKNLKIALKSSYPETYKDLLEIIPGVTMASILIEVVNCVEKIYEAVEEFSGLAHFKETLDSKLS<br>AEIGQHQLLHRGCVKPVLDGDNEKEDNSSCHVL
AtALMT1	MEKVREIVREGIRVGNEDPRRIIHAFKVGLALVLVSSFYYYQPFGPFTDYFGINAMWAVMTVVVVFEFSVGATLGKGLNRGV<br>ATLVAGGLGIGAHQLARLSGATVEPILLVMLVFVQAALSTFVRFFPWVKTKFDYGILIFILTFALISLSGFRDEEIMDLAESRLST<br>VVIGGVSCILISIFVCPVWAGQDLHSLLASNFDTLSHFLQDFGDEYFEAREKGDYKVVEKRKKNLERYKSVLDSKSDEEALAN<br>YAEWEPPHGQFRFRHPWKQYVAVGALLRQCAYRIDALNSYINSDFQIPVDIKKKLETPLRRMSSESGNSMKEMSISLKQMIK<br>SSSSDIHVSNSQAACKSLSTLLKSGILNDVEPLQMISLMTTVSMLIDIVNLTEKISESVHELASAARFKNKMRPTVLYEKSDSGS<br>IGRAMPIDSHEDHHVVTV
TaALMT1	RKVGGAAREDPRRVAHSLKVGLALALVSVVYFVTPLFNGLGVSAIWAVLTVVVVMEYTVGATLSKGLNRALATLVAGCIAV<br>GAHQLAELAERCGDQGEPIVLTVLVFFVASAATFLRFIPEIKAKYDYGVTIFILTFGLVAVSSYRVEELIQLAHQRFYTIAVGVFI<br>CLCTTVFLFPVWAGEDVHKLASGNLDKLAQFIEGMEFNCFGENSVANNFGGKDSPQMHKSVLNSKATEDSLCTFAKWEPR<br>HGQFRFRHPWSQYQKLGTLCRQCASSMEALASYVITTSKTQCPAAANPELSCKVRKTCGEMSLHSSKVLRDLAMATRTMT<br>VPSPVNITMATAVKAAESLRSELAENTALLQVMHVAVTATLLADLVDRVKEIAECVDVLARLAHFKNPEDTKN
AtALMT10	WLKDLVMKVIMERVAKFMRKAWRIGADDPAKVVHCLKVGLALSLVSIFYYMRPLYDGVGGNAMWAIMTVVVVFESNVG

	ATFCKCVNRVVATILAGSLGIAVHWVATQSGKAEVFVIGCSVFLFAFAATYSRFVPSFKARFDYGAMIFILTFSLVSVGGYRVD<br>KLVELAQQRVSTIAIGTSICIIITVFFCPIWAGSQLHRLIERNLEKLADSLDGCVAEYFKENEVSTNRNEDENTNMKLQGFKCV<br>LNSKGTEEAMPLIRFSGFSFSQANLARWEPAHGSFNFRHPWKLYVKIGAAMRRCAYCLENLSICINYETEAPDQVKNHFGE<br>ACMKLSSASSKILRELADMMKNTRKSSKMDFLVFDMNSAVQELQETLKNVPIETNKPEEVPSEEENKVDSEERTTSMSLHEV<br>LPVATLVSLLIENAARIQTAVEAVDELANLADFEQDSKKKTGDNNTKQ
AtALMT14	DRVHERSMGMEEEGSTKNMKTKVLELPTKIKKILKNIWKVGKDDPRRVKHALKVGVSLTLVSLLYLMEPLFKGIGNSAIWA<br>VMTVVVVLEFSAGATLCKGLNRGLGTLIAGSLAFFIEFVANDSGKIFRAIFIGAAVFIIGALITYLRFIPYIKKNYDYGMLIFLLTF<br>NLITVSSYRVDTVIKIAHERFYTIAMGVGICLLMSLLVFPIWSGEDLHKSTVAKLQGLSYSIEACVNEYFEEEEKDEETSDLSED<br>TIYNGYKTVLDSKSADEALAMYASWEPRHTRHCHRFPWKHYVKVGSVLRQFGYTVVALHGCLKTEIQTPRPLRGLFKDPC<br>VRLAGEICKVLSELAASIRN
AtALMT12	MEEGLSKTKWMVLEPSEKIKKIPKRLWNVGKEDPRRVIHALKVGLSLTLVSLLYLMEPLFKGIGSNAIWAVMTVVVVLEFSA<br>GATLCKGLNRGLGTLIAGSLAFFIEFVANDSGKVLRAIFIGTAVFIIGAAATYIRFIPYIKKNYDYGVVIFLLTFNLITVSSYRVDS<br>VINIAHDRFYTIAVGCGICLFMSLLVFPIWSGEDLHKTTVGKLQGLSRSIEACVDEYFEEKEKEKTDSKDRIYEGYQAVLDSKS<br>TDETLALYANWEPRHTLRCHRFPCQQYVKVGAVLRQFGYTVVALHGCLQTEIQTPRSVRALFKDPCVRLAGEVCKALTELA<br>DSI
AtALMT13	KGLNLPKKMKKILRNLWNVGKEDPRRVIHALKVGVALTLVSLLYLMEPFFEGVGKNALWAVMTVVVVLEFSAGATLRKGL<br>NRGLGTLIAGSLAFFIEWVAIHSGKILGGIFIGTSVFTIGSMITYMRFIPYIKKNYDYGMLVFLLTFNLITVSSYRVDTVIKIAHER<br>LYTIGMGIGICLFMSLLFFPIWSGDDLHKSTITKLQGLSRCIEACVSEYFEEKLKDNETSDSESDDEDLIYNGYNTVLDSKSADE<br>ALAMYAKWEPRHTRRCNKFPSQQYIKVGSVLRKFGYTVVALHGCLQTEIQTPRSIRVLFKDPCVRLAGEICKVLSELSESIQN
AtALMT9	LSEKISGVYDDAKDVARKAWEMGVSDPRKIVFSAKIGLALTIVALLIFYQEPNPDLSRYSVWAILTVVVVFEFTIGATLSKGFN<br>RALGTLSAGGLALGMAELSTLFGDWEEIFCTLSIFCIGFLATFMKLYPSMKAYEYGFRVFLLTYCYILISGFRTGQFIEVAISRFLL<br>IALGAGVSLGVNMFIYPIWAGEDLHNLVVKNFMNVATSLEGCVNGYLRCLEYERIPSKILTYQASEDPVYKGYRSAVESTSQE<br>ESLMSFAIWEPPHGPYKSFNYPWKNYVKLSGALKHCAFTVMALHGCILSEIQAPEERRQVFRQELQRVGVEGAKLLRELGE<br>KVKKM
AtALMT3	KITGVVKKLKDVLVTAWEMGTADPRKMIFSAKMGLALTLTSILIFFKIPGLELSGHYLWAILTVVVIFEFSIGATFSKGCNRGLG<br>TLSAGGLALGMSWISEMTGNWADVFNAASIFVVAFFATYAKLYPTMKPYEYGFRVFLLTYCYVIVSGYKTGEFMETAVSRFLL<br>IALGASVGLIVNTCIYPIWAGEDLHNLVAKNFVNVATSLEGCVNGYLECVAYDTIPSRILVYEAVAEDPVYSGYRSAVQSTSQE

	DTLMSFASWEPPHGPYKSFRYPWALYVKVGGALRHCAIMVMALHGCILSEIQAAEDRRREFRNELQRVGIEGAKVLRYIGESLKKM
AtALMT6	ELGHSDRRRIFFAVKMGMALALCSVVIFLKEPLHDASKYSVWGILTVVVVFEYSVGATLVKGFNRAIGTVSAGGLALGIARLSVLSRDFEQTIIITCIFLAGFIASYSKLHPAMKPYEYAFRVFLLTFCIVLVSGNNTGDFFSTAYYRFLFIVVGATTCLVVNIFIFPIWAGEDLHKLVANNFKSVANSLEGCVNGYLQCVEYERVPSKILTYQTSDDPLYSGYRSAIQSTNQEESLLDFAIWEPPHGPYRTFNHPWKNYVKLSGAVRHCAFTVMAIHGCILSEIQAAPEKRQAFRHELQRVGNEGAKVLRLIGEKVEKM
AtALMT4	SDGITASWKALYDIGAKLYEMGRSDRRKVYFSVKMGMALALCSFVIYLKEPLRDASKYAVWAILTVVVVFEYSIGATLVKGFNRAIGTLSAGGLALGIARLSVSAGEFEELIIIISIFIAGFSASYLKLYPAMKSYEYAFRVFLLTYCIVLVSGNNSRDFFSTAYYRFLLILVGAGICLGVNIFILPIWAGEDLHKLVVKNFKSVANSLEGCVNGYLQCVEYERIPSKILTYQASDDPLYSGYRSVVQSTSQEDSLLDFAVWEPPHGPYKTFHHPWANYVKLSGAVRHCAFMVMAMHGCILSEIQAAPEKRQAFRQELQRVGNEGAKVLRLFGEKVEKM
AtALMT5	KLYALGHSDRRKLYFSIKMGIALALCSFVIFLKEPLQDASKFAVWAILTVVLIFEYYVGATLVKGFNRALGTMLAGGLALGVAQLSVLAGEFEEVIIVICIFLAGFGASYLKLYASMKPYEYAFRVFKLTYCIVLVSGNNSRDFLSTAYYRILLIGLGATICLLVNVFLFPIWAGEDLHKLVAKNFKNVANSLEGCVNGYLQCVEYERIPSKILTYQASDDPLYSGYRSAVQSTSQEDSLLDFAIWEPPHGPYKTFNHPWKNYVKLSGAVRHCAFTVMAMHGCILSEIQASPEKRHVFSNELRRVGNEGAKVLRLFGEKVEKM
AtALMT11	LSKTKWMVLEPSEKIKKIPKRLWSVGKEDPRRVIHAFKVGHSLTLVSLLYFMENLFKGIGSNAIWAVMTVVAVLLEF
